# Development of high-yield influenza A virus vaccine viruses

**DOI:** 10.1038/ncomms9148

**Published:** 2015-09-02

**Authors:** Jihui Ping, Tiago J.S. Lopes, Chairul A. Nidom, Elodie Ghedin, Catherine A. Macken, Adam Fitch, Masaki Imai, Eileen A. Maher, Gabriele Neumann, Yoshihiro Kawaoka

**Affiliations:** 1 Department of Pathobiological Sciences, School of Veterinary Medicine, Influenza Research Institute, University of Wisconsin-Madison, Madison, Wisconsin 53711, USA; 2 Division of Virology, Department of Microbiology and Immunology and International Research Center for Infectious Diseases, The Institute of Medical Science, University of Tokyo, Tokyo 108-8639, Japan; 3 Avian Influenza-Zoonosis Research Center, Airlangga University, Surabaya 60115, Indonesia; 4 Department of Biology, New York University, New York, New York 10003 USA; 5 Department of Computational & Systems Biology, University of Pittsburgh School of Medicine, Pittsburgh, Pennsylvania 15261 USA; 6 Bioinformatics Institute, University of Auckland, Auckland 1010, New Zealand

## Abstract

Vaccination is one of the most cost-effective ways to prevent infection. Influenza vaccines propagated in cultured cells are approved for use in humans, but their yields are often suboptimal. Here, we screened A/Puerto Rico/8/34 (PR8) virus mutant libraries to develop vaccine backbones (defined here as the six viral RNA segments not encoding haemagglutinin and neuraminidase) that support high yield in cell culture. We also tested mutations in the coding and regulatory regions of the virus, and chimeric haemagglutinin and neuraminidase genes. A combination of high-yield mutations from these screens led to a PR8 backbone that improved the titres of H1N1, H3N2, H5N1 and H7N9 vaccine viruses in African green monkey kidney and Madin–Darby canine kidney cells. This PR8 backbone also improves titres in embryonated chicken eggs, a common propagation system for influenza viruses. This PR8 vaccine backbone thus represents an advance in seasonal and pandemic influenza vaccine development.

## Abstract (web summary)

The availability of high-yield virus strains remains an important bottleneck in the rapid production of influenza vaccines. Here, the authors report the development of influenza A vaccine backbone that improves the virus yield of various seasonal and pandemic influenza vaccine strains in cell culture.

## Graphical abstract

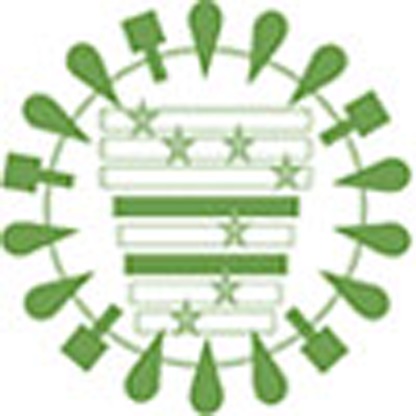

Influenza A viruses belong to the family *Orthomyxoviridae* with a genome composed of eight single-stranded, negative-sense viral RNA segments. Based on the antigenicity of the two major surface antigens, haemagglutinin (HA) and neuraminidase (NA), influenza A viruses are currently categorized into 18 HA subtypes (H1–H18) and 11 NA subtypes (N1–N11). Each year, seasonal influenza A viruses of the H1N1 and H3N2 subtypes and influenza B viruses cause several hundred million human infections and 250,000 to 500,000 deaths worldwide (http://www.who.int/mediacentre/factsheets/fs211/en/). Occasionally, reassortment of viral RNA segments results in novel strains to which most humans are naïve, such as during the pandemics of 1918, 1957, 1968 and 2009 (reviewed in ref. 1). In addition, sporadic infections of humans with avian influenza A viruses of the H5N1 or H7N9 subtypes (resulting in case fatality rates of ∼60 and ∼30%, respectively) (http://www.who.int/influenza/human_animal_interface/EN_GIP_20140124CumulativeNumberH5N1cases.pdf?ua=1)^2^,^3^ have been a major public health concern.

Due to frequent ‘antigenic drift' (that is, the accumulation of point mutations in the antigenic epitopes of HA) and occasional ‘antigenic shift' (that is, the introduction into human populations of an HA to which humans are immunologically naive), new influenza A vaccine strains are recommended every ∼1–5 years by the World Health Organization (WHO). For the generation of inactivated vaccines, the HA and NA viral RNA segments of the recommended strains are typically combined with the remaining six viral RNA segments of A/Puerto Rico/8/34 (PR8; H1N1) virus by using reassortment or reverse genetics approaches. Most vaccine viruses are then propagated in embryonated chicken eggs. However, this widely used influenza vaccine production platform has several vulnerabilities, including the potential for the egg supply to become a limiting factor for rapid large-scale vaccine production in the event of an influenza pandemic. In addition, virus propagation in embryonated chicken eggs frequently results in egg-adapting mutations in HA that can affect the antigenicity of the virus^4^,^5^,^6^,^7^,^8^,^9^,^10^,^11^ and may be responsible for the reduced efficacy of some influenza vaccines^12^,^13^.

Mammalian cell culture-based vaccine production has distinct advantages over the currently prevalent egg-based production system: it is more easily scaled up and has reduced risk for the emergence of mutations that result in antigenic changes^14^. As an added advantage, the absence of egg proteins in cell culture-based vaccines eliminates potential complications for individuals with egg allergies^15^,^16^. To date, influenza vaccines produced in African green monkey kidney (Vero) and Madin–Darby canine kidney (MDCK) cells^17^,^18^ have been approved for human use in the United States and/or some European countries.

Vaccine virus yield is a critical parameter in the vaccine manufacturing process. Although the PR8 backbone generally confers efficient replication in embryonated chicken eggs, occasionally PR8-based vaccines have low yields, leading to delays in vaccine manufacturing. Examples are the original A/Fujian/411/2002 (H3N2)^19^ and 2009 pandemic H1N1^20^,^21^ vaccine candidates, which replicated poorly in embryonated chicken eggs. In addition, lower virus yields in cultured cells compared with embryonated chicken eggs may have hampered the wider use of cell culture-based influenza vaccine production platforms^18^. Therefore, efforts have been undertaken to optimize the production parameters and the vaccine viruses. Sequential passages of vaccine viruses in embryonated chicken eggs can improve virus yield in an egg-based vaccine production system (reviewed in refs 20, 21). However, passaging in eggs adds to the production time, must be repeated for any low-producing strain, and frequently leads to mutations in HA that may affect viral antigenicity, as stated earlier. Some studies have attempted to improve the replication efficiency of vaccine viruses by using reassortant viruses or viruses encoding chimeric or mutated HA and/or NA proteins^22^,^23^,^24^,^25^,^26^,^27^; to date, none of these studies has resulted in commercially available, improved influenza vaccine production. An alternative strategy for boosting vaccine production is to modify the virus backbone (that is, PR8) for increased replication efficiency. This approach has the potential to work in a strain-independent manner, avoiding time required for preliminary adaptation of a new strain to the culture system. We therefore carried out a comprehensive study to develop a PR8 vaccine backbone that significantly improves the virus yield of various seasonal and pandemic influenza vaccines strains in cell culture. As we show, the same backbone can also improve yield in egg culture systems.

## Results

### Virus library screens for high-yield variants

**Figure 1:**
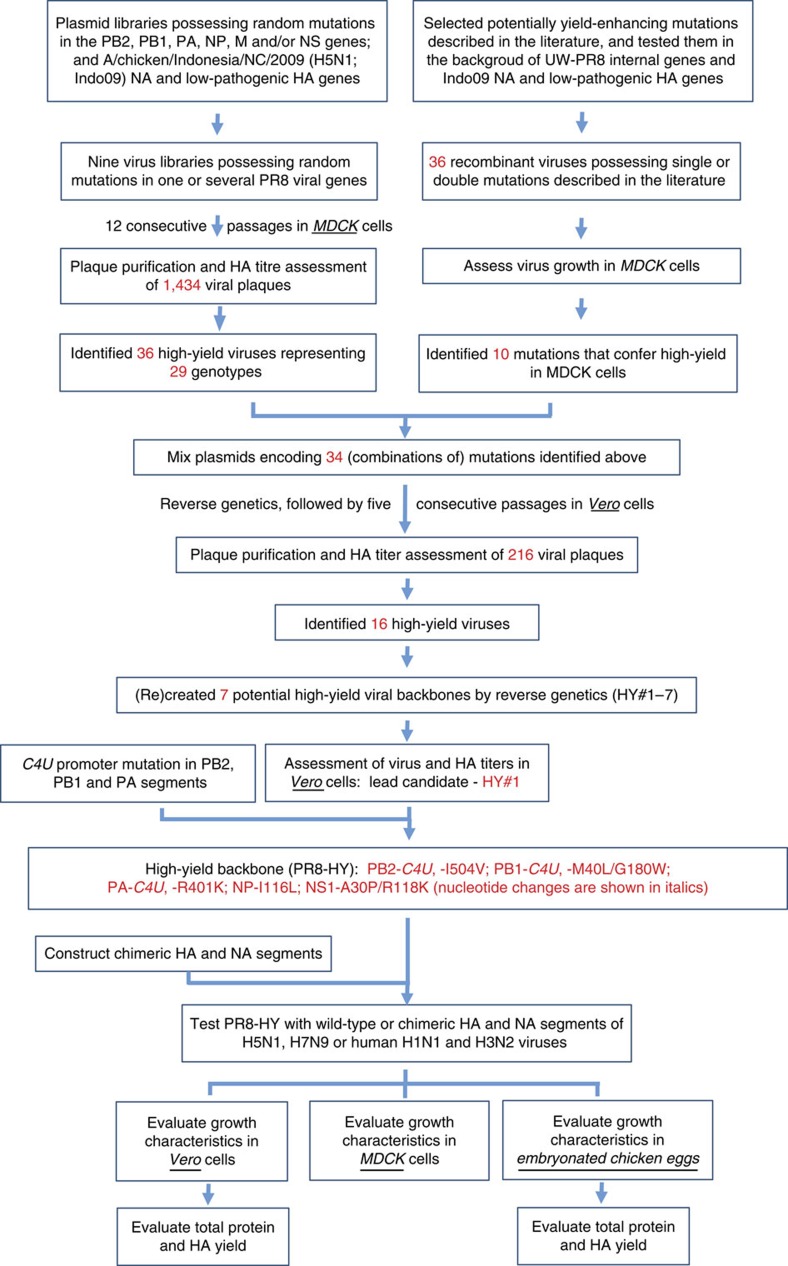
Flow chart summarizing the selection and testing of PR8-HY. Details are described in the text.

Currently, it is not known which mutations in PR8 increase the viruses' replicative ability in cultured cells. Therefore, we used a high-throughput mutagenesis and screening approach to identify mutations associated with high yield of PR8. Specifically, we used error-prone PCR to generate large sets (that is, ‘libraries') of viral cDNAs with random mutations (see Methods for details). Using reverse genetics approaches, these mutant cDNA libraries were used to generate virus libraries composed of tens of thousands of variants possessing random amino-acid changes in one or several viral proteins. Based on UW-PR8 (a high-growth variant of PR8 maintained at the University of Wisconsin-Madison^28^,^29^), we generated the following nine virus libraries: six libraries possessing random mutations in each of the ‘internal' genes (that is, PB2, PB1, PA, NP, M and NS); one library possessing random mutations in the genes encoding the PB2, PB1 and PA proteins, which together form the viral polymerase complex; one library possessing random mutations in the PB2 and NS genes, which code for the major virulence factors PB2 and NS1, respectively (reviewed in^1^,^30^); and one library possessing random mutations in the NS and M genes since the M1 protein (encoded by the M gene) is associated with high-growth properties^31^ (Fig. 1). Since high-yield vaccine backbones are urgently needed for vaccines to pandemic H5N1 viruses, we combined the HA and NA genes of an H5N1 virus, A/chicken/Indonesia/NC/2009 (Indo09; H5N1; subclade 2.1.3.2) with the UW-PR8 backbone for our library generation and screening procedure. We replaced the multibasic sequence at the Indo09 HA cleavage site with a single basic amino acid so that the viruses generated with the PR8 backbone were of low pathogenicity, and therefore exempt from Select Agent status by the United States Animal and Plant Health Inspection Service (APHIS) and approved for studies at BSL-2.

To select high-yield variants, each of the nine libraries of viruses composed of a mutant UW-PR8 backbone with HA and NA from Indo09 was passaged 12 times in MDCK cells; in parallel, these nine libraries were combined after the second passage, and then passaged a further 10 times in MDCK cells (Supplementary Fig. 1). From these experiments, we randomly selected ∼100–150 plaques per library, resulting in a total of 1434 individual, plaque-purified viruses (Fig. 1). The yield of each plaque-purified virus was compared in haemagglutination (HA) assays with the yield of a control virus composed of an unmutated UW-PR8 backbone and the Indo09 HA/NA genes (UW-PR8_Indo09). We identified 36 viruses with HA titres that were at least twofold higher than that of the control virus. These high-yield candidates were ‘purified' through an additional round of plaque assays, followed by virus amplification in MDCK cells. To identify the mutations responsible for the increased replicative ability of the 36 high-yield candidates in MDCK cells, we sequenced their entire genomes. Twenty-nine different combinations of mutations were detected (Fig. 1 and Supplementary Table 1). The most frequently observed amino-acid changes localized to the polymerase subunits PB2 (namely, PB2-M202L/F323L and PB2-I504V) and PB1 (namely, PB1-V644A and PB1-E112G; note that the nucleotide change causing the PB1-E112G mutation also causes an R81G mutation in the overlapping PB1-F2 protein). Although the HA and NA genes were not targeted by PCR-mediated mutagenesis, several mutations were detected in these proteins, likely reflecting adaptation of the Indo09 HA and NA proteins to MDCK cells. None of the mutations in HA restored the multibasic sequence at the HA cleavage site.

### Testing of mutations identified in the literature

Multiple amino-acid changes have been associated with altered replication kinetics of a variety of viruses in mammalian systems (Supplementary Table 2). The effect of most of these mutations on UW-PR8 in mammalian systems is unknown. We, therefore, used site-directed mutagenesis to generate 36 mutant UW-PR8_Indo09 viruses, each carrying a mutation(s) selected from Supplementary Table 2, and assessed their growth kinetics in MDCK cells (Fig. 1 and Supplementary Fig. 2). Ten mutants exhibited significant increases in virus yield compared with the control virus UW-PR8_Indo09 (Supplementary Fig. 2 and Supplementary Table 3). One of these mutants (carrying PB2-I504V and PA-I550L) was also isolated from our randomly mutated virus libraries (see Supplementary Table 1), suggesting that it confers high-yield properties to UW-PR8_Indo09.

### Potential combinatorial effects of mutations

On the basis of our library screens and the systematic testing of mutations identified through literature searches, we generated 34 mutants for further study. Thirty-two of these carried single or double mutations selected from those that caused the greatest increases in yield and/or were found most frequently (Fig. 1 and Supplementary Table 4). In addition, the NP-R422K mutation was selected because of its role in NP oligomer formation^32^, although it did not confer a statistically significant increase in virus titres (Supplementary Fig. 2 and Supplementary Table 3). Similarly, the NS1-K55E mutation was selected for its demonstrated contribution to the high-growth properties of UW-PR8 virus^29^, although it did not increase virus titres here (Supplementary Fig. 2 and Supplementary Table 3). To test whether combinations of these mutations would further increase the growth properties of UW-PR8_Indo09, we transfected 293T cells with eight RNA polymerase I plasmids encoding the wild-type viral RNA segments, and with 34 RNA polymerase I plasmids encoding the mutations shown in Supplementary Table 4. To initiate viral RNA transcription and replication, these cells were also transfected with four plasmids encoding the A/WSN/33 (H1N1) polymerase and NP proteins. In total, the cells were co-transfected with 46 different plasmids. This strategy should result in viruses encoding all candidate high-yield mutations shown in Supplementary Table 4.

Ideally, a backbone for cell-based vaccine production will confer increased growth properties in both MDCK and Vero cells. We therefore passaged the above-described virus mixture five times in Vero cells and isolated 216 individual viruses, which were assayed for HA titres (Fig. 1). Sixteen viruses exhibited HA titres of 2^9–9.5^, compared with an HA titre of 2^6.5^ for the control UW-PR8_Indo09 virus (Fig. 1 and Supplementary Table 5). Sequence analysis of these high-yield candidates revealed dominant mutations in several viral proteins (Supplementary Table 5).

**Figure 2:**
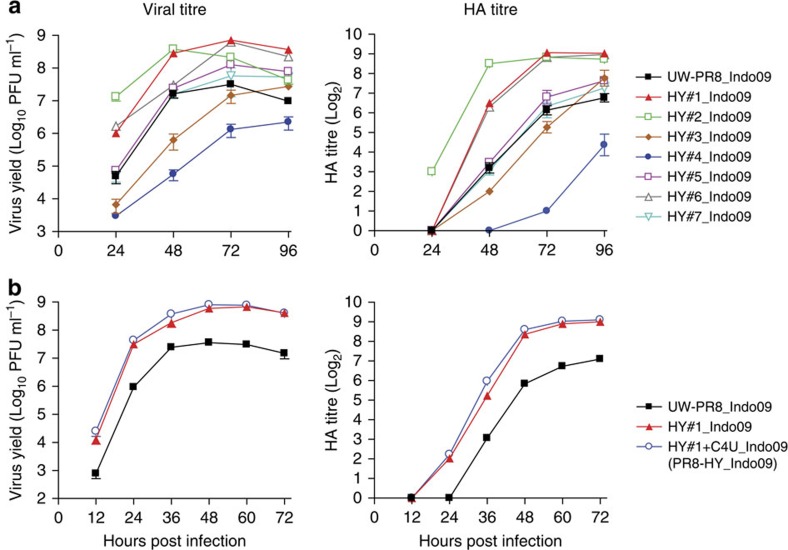
Growth kinetics and HA titres of HY#1–7 high-yield candidates in Vero cells. (**a**) Growth kinetics and HA titres of high-yield candidates in Vero cells. Vero cells were infected in triplicate with the indicated viruses at a multiplicity of infection (MOI) of 0.005 and incubated at 37 °C. Supernatants were collected at the indicated time points, and the virus titres were determined by plaque assays in MDCK cells. In parallel, we determined the HA titres of the collected supernatants by performing HA assays. (**b**) Effect of the C4U promoter mutation in the viral polymerase genes on viral growth kinetics and HA titres. Shown is the comparison of viruses possessing the parental UW-PR8 backbone (UW-PR8_Indo09), the HY#1 backbone (HY#1_Indo09), or the HY#1 backbone with C4U mutations in the PB2, PB1 and PA genes (HY#1+C4U_Indo09). Experiments were carried out as described in **a**. The values presented are the average of three independent experiments±s.d.

To confirm the yield-enhancing effects of the amino-acid changes in these 16 selected viruses, we used reverse genetics to make seven different mutant UW-PR8_Indo09 viruses, referred to as HY#1–7_Indo09 (Supplementary Table 6). Each of these mutant viruses carries a different combination of amino-acid changes selected from those carried by the 16 high-yield viruses. Six of these mutants exhibited significantly higher HA and/or virus titres in Vero cells than the control UW-PR8_Indo09 virus (Fig. 2a and Supplementary Table 7). The highest titres were detected for HY#1_Indo09, which carries the PB2-I504V, PB1-M40L/G180W, PA-R401K, NP-I116L and NS1-A30P/R118K mutations; the HY#1 vaccine backbone was therefore selected for further studies.

### Mutations in the influenza promoter and non-coding regions

The influenza A viral promoter sequences, that is, the 12 and 13 nucleotides at the 3′ and 5′ termini of the vRNAs, respectively, are highly conserved among the eight viral RNA segments as well as among viruses. Mutations in these regions can significantly affect the viral polymerase activity in reporter gene assays^33^,^34^. We, therefore, tested 20 UW-PR8_Indo09 virus libraries that possessed various mutations at several positions in the promoter region of the HA gene; however, we were unable to select mutants with increased replicative ability in MDCK cells.

The viral promoters are separated from the start and stop codons of the viral open reading frames by a variable number of nucleotides, depending on the viral RNA segment. These so-called ‘non-coding regions' differ among the viral RNA segments and among virus strains. Here, we tested 11 virus libraries in which portions of the non-coding region of the UW-PR8_Indo09 HA segment were randomized; these mutations did not increase the replicative ability of UW-PR8_Indo09 virus in MDCK cells.

Our random mutations of the promoter region did not affect virus yield in MDCK cells. Nevertheless, potentially significant variability in the fourth position from the 3′ end of the viral RNAs, where a C or U (C4 or U4) residue is found, has been reported^35^. Specifically, in MDCK cells, the titre (assessed by using plaque assays) of a A/WSN/33 virus encoding U4 in all eight viral RNA segments was more than two log_10_ units higher than that of a control A/WSN/33 virus encoding C4 (ref. 35). We, therefore, converted the C4 residues found in the three polymerase genes of HY#1 to U4, resulting in HY#1+C4U_Indo09, which replicated to slightly higher titres in Vero cells compared with HY#1_Indo09 (Fig. 2b and Supplementary Table 7); HY#1+C4U_Indo09 was therefore selected as the final high-yield backbone in Vero cells, and was designated PR8-HY (Fig. 1). Compared with the UW-PR8 backbone, the PR8-HY backbone possesses the following mutations: *PB2-C4U* (nucleotide changes are shown in italics), PB2-I504V, *PB1-C4U*, PB1-M40L/G180W, *PA-C4U*, PA-R401K, NP-I116L and NS1-A30P/R118K (Fig. 1).

### Chimeric HA and NA genes increase virus yield

**Figure 3:**
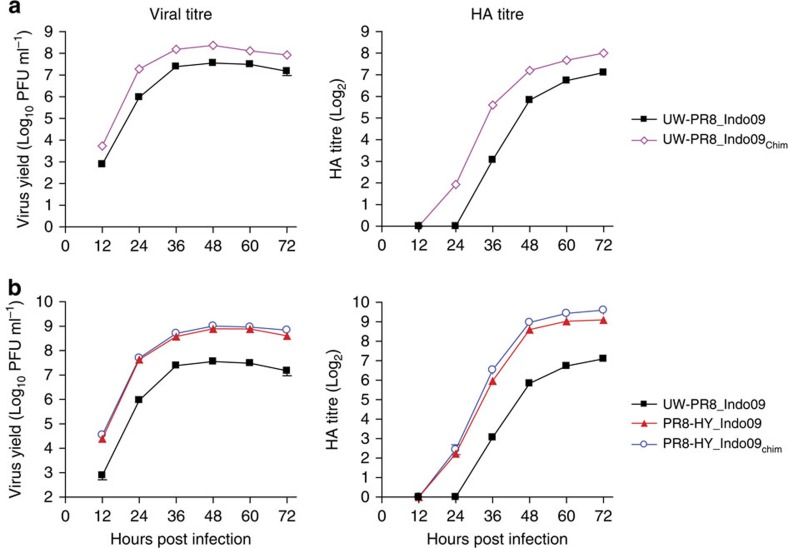
Growth kinetics and HA titres of HA/NA chimeric viruses in Vero cells. (**a**) Growth kinetics and HA titres of UW-PR8-based viruses with wild-type or chimeric Indo09 HA and NA genes. (**b**) Growth kinetics and HA titres of viruses possessing the UW-PR8 backbone in combination with Indo09 HA and NA genes, or the PR8-HY backbone in combination with wild-type or chimeric Indo09 HA and NA genes. Experiments were carried out as described in the legend to Fig. 2. The values presented are the average of three independent experiments±s.d.

In parallel to the development of a UW-PR8-based high-yield backbone, we also tested chimeric HA and NA genes/proteins, a strategy that has been shown to increase virus yield^22^,^23^,^24^,^25^,^26^,^27^. In these chimeras, the extracellular domains of HA and NA are derived from the recommended vaccine strain, whereas the transmembrane and intracellular domains originate from PR8 viruses to ensure optimal compatibility with the internal, PR8 virus-derived viral genes and proteins. We, therefore, generated a UW-PR8_Indo09_Chim_ virus in which only the extracellular domains of the HA and NA proteins were derived from Indo09 virus (see Supplementary Fig. 3 and Supplementary Table 8). In Vero cells, the virus and HA titres of this virus were significantly higher than those of the UW-PR8_Indo09 virus, which encodes the authentic Indo09 HA and NA genes (Fig. 3a and Supplementary Table 9). Therefore, we next tested the chimeric Indo09 HA and NA genes with the high-yield backbone (PR8-HY), resulting in PR8-HY_Indo09_Chim_. We detected a small, but statistically significant increase in virus titres for PR8-HY_Indo09_Chim_ relative to those for PR8-HY_Indo09 (Fig. 3b and Supplementary Table 9).

### Evaluation of candidate vaccine viruses in Vero cells

**Figure 4:**
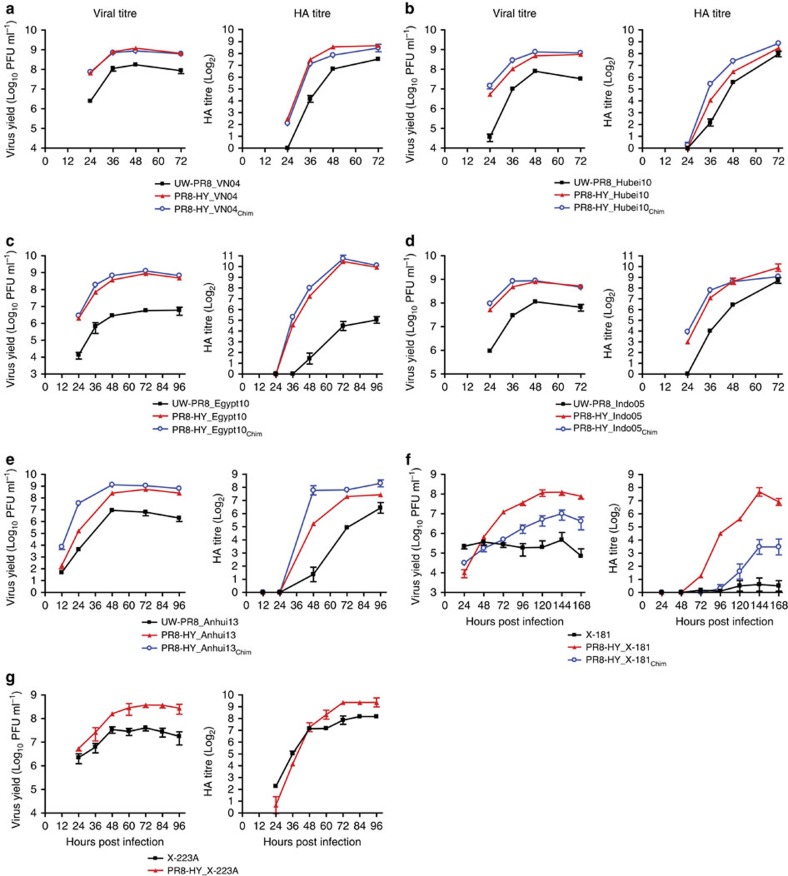
Evaluation of PR8-HY backbone vaccine candidates propagated in Vero cells. Growth kinetics and HA titres of UW-PR8- and PR8-HY-based viruses encoding the wild type, or wild-type and chimeric HA and NA segments of the A/Vietnam/1203/2004 (VN04, H5N1) (**a**), A/Hubei/1/2010 (Hubei10, H5N1) (**b**), A/Egypt/N03072/2010 (Egypt10, H5N1) (**c**), A/Indonesia/5/2005 (Indo05, H5N1) (**d**) or A/Anhui/1/2013 (Anhui13, H7N9) (**e**) viruses. Panels (**f**) and (**g**) show a comparison of current seasonal H1N1 and H3N2 vaccine viruses (X-181 and X-223A, respectively) with PR8-HY backbone viruses possessing wild-type or chimeric HA and NA segments derived from X-181 or X-223A viruses. Experiments were carried out as described in the legend to Fig. 2, with the exception of those involving X-181 and X-223A viruses, which were inoculated at an MOI of 0.1. The values presented are the average of three independent experiments±s.d.

Ideally, the virus backbone used for influenza vaccine production should yield high virus and HA titres with the HA and NA genes of varied seasonal and pandemic influenza viruses. Accordingly, we first tested the PR8-HY backbone with wild-type or chimeric HA and NA genes of different WHO-recommended H5N1 vaccine viruses, namely, A/Vietnam/1203/2004 (Clade 1; VN04), A/Hubei/1/2010 (Clade 2.3.2.1a; Hubei10), A/Egypt/N03072/2010 (Clade 2.2.1; Egypt10), and A/Indonesia/05/2005 (Clade 2.1.3.2; Indo05); all H5 HA genes were mutated to encode an HA cleavage sequence characteristic of low pathogenic viruses. We tested titres up to 72 h post infection, except for the inefficiently replicating Egypt10 virus, which was also tested at 96 h post infection (Fig. 4a–d). The PR8-HY backbone increased virus and HA titres significantly compared with the respective control viruses possessing the UW-PR8 backbone (Fig. 4a–d and Supplementary Table 10). The use of chimeric HA and NA genes provided a statistically significant increase in virus and/or HA titres for Hubei10, Egypt10 and Indo05, but not for VN04 viruses (Fig. 4a–d and Supplementary Table 10). Together, these improvements increased virus titres ∼5−220-fold compared with the respective UW-PR8 viruses (Table 1), demonstrating the potential of the PR8-HY backbone as vaccine vector.

Next, we used the same strategy to test the PR8-HY backbone with the wild-type or chimeric HA and NA genes of A/Anhui/1/2013 (H7N9) virus, a representative of the recently emerged H7N9 viruses that have caused severe respiratory infections in humans with high-case fatality rates^2^,^3^. The Vero cell titres of PR8-HY viruses expressing wild-type or chimeric A/Anhui/1/2013 HA and NA genes were 85-fold and 173-fold higher, respectively, than those of the UW-PR8-based control virus (Table 1 and Fig. 4e). A significant increase in HA titres was found in conjunction with this significant increase in virus titres (Fig. 4e and Supplementary Table 10).

Finally, we tested the PR8-HY backbone with the wild-type and chimeric HA and NA genes of the current human H1N1 and H3N2 vaccine viruses, New York Medical Center (NYMC) X-181 (X-181) and NYMC X-223A (X-223A), respectively. X-181, which possesses the A/California/07/2009 (H1N1; CA09) HA, NA and PB1 genes and the remaining genes from of the WHO-recommended PR8 virus, did not replicate to high titres, even after an extended period of replication (Fig. 4f); by contrast, the PR8-HY backbone with the CA09 HA and NA genes increased virus titres >250-fold (Fig. 4f, Table 1 and Supplementary Table 10); hence, our high-growth vaccine candidate confers significantly higher virus titres than the NYMC PR8 backbone currently used for the generation of seasonal influenza vaccines. Interestingly, chimeric HA and NA genes had a strongly attenuating effect on this vaccine candidate (Fig. 4f). The current human seasonal H3N2 vaccine virus is based on the A/Texas/50/2012 HA and NA genes combined with the remaining genes of the WHO-recommended PR8 isolate. This virus replicates to moderate titres in Vero cells, which can be improved significantly with the use of the PR8-HY backbone (Fig. 4g and Supplementary Table 10). Notably, we were unable to generate a PR8-HY virus possessing chimeric X-223A-derived HA and NA genes. Thus, for both seasonal human vaccine viruses tested here, chimeric HA and NA genes were attenuating, rather than titre enhancing.

### Evaluation of candidate vaccine viruses in MDCK cells

**Figure 5:**
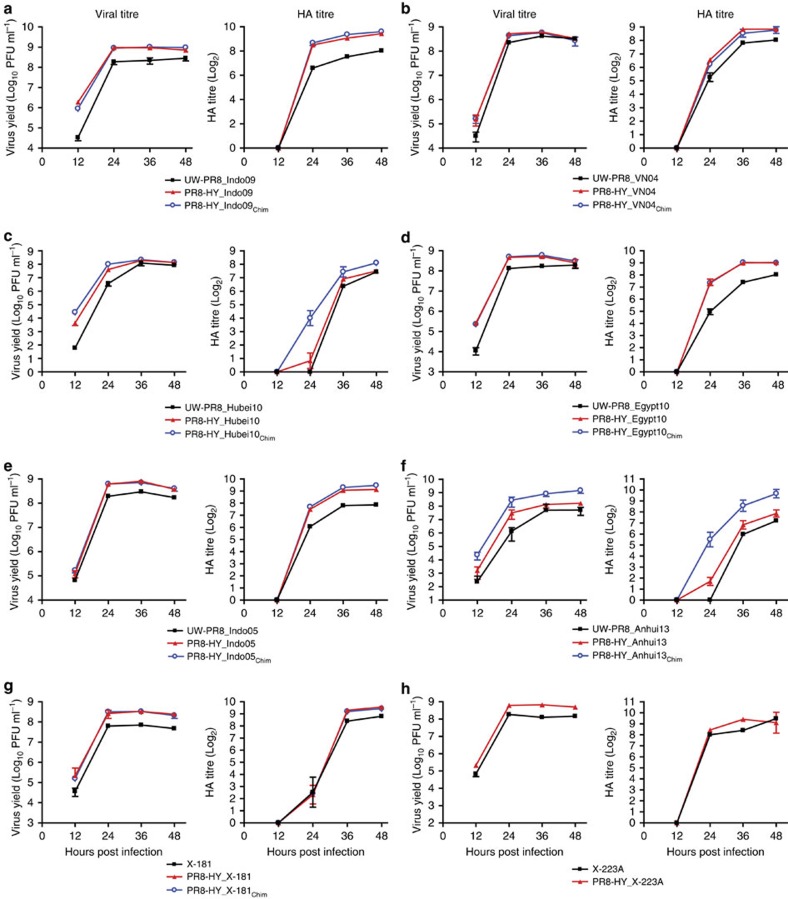
Evaluation of PR8-HY vaccine candidate viruses propagated in MDCK cells. Growth kinetics and HA titres of UW-PR8- and PR8-HY-based viruses encoding the wild-type, or wild-type and chimeric HA and NA segments of the A/chicken/Indonesia/NC/2009 (Indo09, H5N1) (**a**), A/Vietnam/1203/2004 (VN04, H5N1) (**b**), A/Hubei/1/2010 (Hubei10, H5N1) (**c**), A/Egypt/N03072/2010 (Egypt10, H5N1) (**d**), A/Indonesia/5/2005 (Indo05, H5N1) (**e**), or A/Anhui/1/2013 (Anhui13, H7N9) (**f**) viruses. Panels (**g**) and (**h**) show a comparison of current seasonal H1N1 and H3N2 vaccine viruses (X-181 and X-223A, respectively) with PR8-HY backbone viruses possessing wild-type or chimeric HA and NA segments derived from X-181 or X-223A viruses. MDCK cells were infected in triplicate with the indicated viruses at an MOI of 0.001 and incubated at 37 °C; otherwise, experiments were carried out as described in the legend to Fig. 2. The values presented are the average of three independent experiments±s.d.

After demonstrating that the PR8-HY backbone confers high yield to pandemic and seasonal influenza vaccine candidates in Vero cells, we performed similar studies in MDCK cells. Based on the efficient growth kinetics of the tested viruses in MDCK cells, titres were determined for up to 48 h post infection (Fig. 5). For all viruses tested, the PR8-HY backbone conferred higher virus and HA titres compared with the UW-PR8 backbone, although not all differences were statistically significant (Fig. 5 and Supplementary Table 11). Chimeric HA and NA genes further improved the virus and/or HA titres of some viruses, namely, PR8-HY_Anhui_Chim_ and PR8-HY_Hubei10_Chim_ (Fig. 5 and Supplementary Table 11). In combination, the PR8-HY backbone and chimeric HA and NA genes increased virus titres by 1.4−29-fold, and HA titres by 1.3−5.5-fold (Table 1).

### Evaluation of candidate vaccine viruses in embryonated eggs

**Figure 6:**
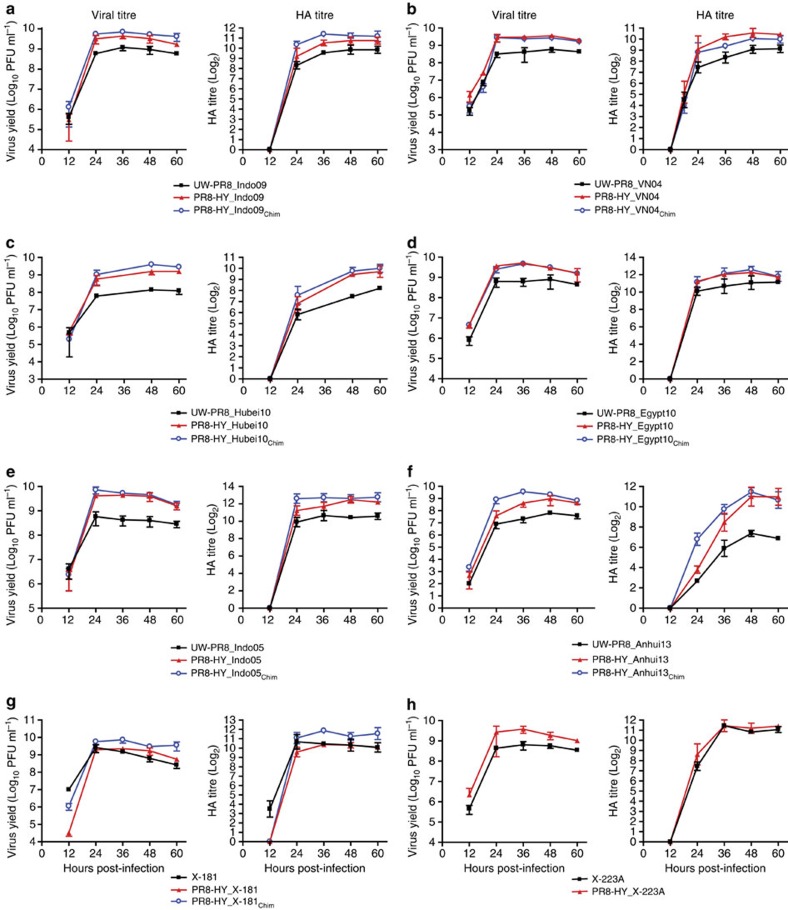
Evaluation of PR8-HY vaccine candidate viruses propagated in embryonated chicken eggs. Growth kinetics and HA titres of UW-PR8- and PR8-HY-based viruses encoding the wild-type, or wild-type and chimeric HA and NA segments of the A/chicken/Indonesia/NC/2009 (Indo09, H5N1) (**a**), A/Vietnam/1203/2004 (VN04, H5N1) (**b**), A/Hubei/1/2010 (Hubei10, H5N1) (**c**), A/Egypt/N03072/2010 (Egypt10, H5N1) (**d**), A/Indonesia/5/2005 (Indo05, H5N1) (**e**), or A/Anhui/1/2013 (Anhui13, H7N9) (**f**) viruses. Panels (**g**) and (**h**) show a comparison of current seasonal H1N1 and H3N2 vaccine viruses (X-181 and X-223A, respectively) with PR8-HY backbone viruses possessing wild-type or chimeric HA and NA segments derived from X-181 or X-223A viruses. Ten-day-old embryonated chicken eggs (four per virus) were inoculated with 2 × 10^3^ PFU of the respective viruses and incubated at 35 °C for the indicated periods of time. The values presented are the average of three independent experiments±s.d.

Although Vero and MDCK cell lines are now available to propagate influenza vaccines for human use, embryonated chicken eggs remain the most commonly used propagation system, prompting us to test our PR8-HY-based vaccine candidates in eggs. As shown in Fig. 6 and Supplementary Table 12, the PR8-HY backbone significantly increased virus and/or HA titres of the candidate vaccines, and the use of chimeric HA and NA genes further increased the titres of some viruses, such as Anhui13. The combined effect of the PR8-HY backbone and chimeric HA and NA genes increased virus titres by 4.6−172-fold, resulting in titres of >10^9^ plaque-forming units (PFU) ml^−1^ (Table 1); HA titres increased by 1.3−17.7-fold (Table 1). Relatively small increases in HA titres were detected for the human seasonal PR8-HY_X-181_Chim_ and PR8-HY_X-223A viruses compared with the parental X-181 and X-223A viruses. X-181 and X-223A were selected as vaccine viruses because of their efficient replication in embryonated chicken eggs (resulting in HA titres of up to 2^11^ Log_2_), so further increases may be difficult to achieve. Collectively, however, our data demonstrate that the Vero cell-optimized PR8-HY vaccine backbone increases vaccine virus titres in embryonated chicken eggs.

### Evaluation of total viral protein yield and HA content

**Figure 7:**
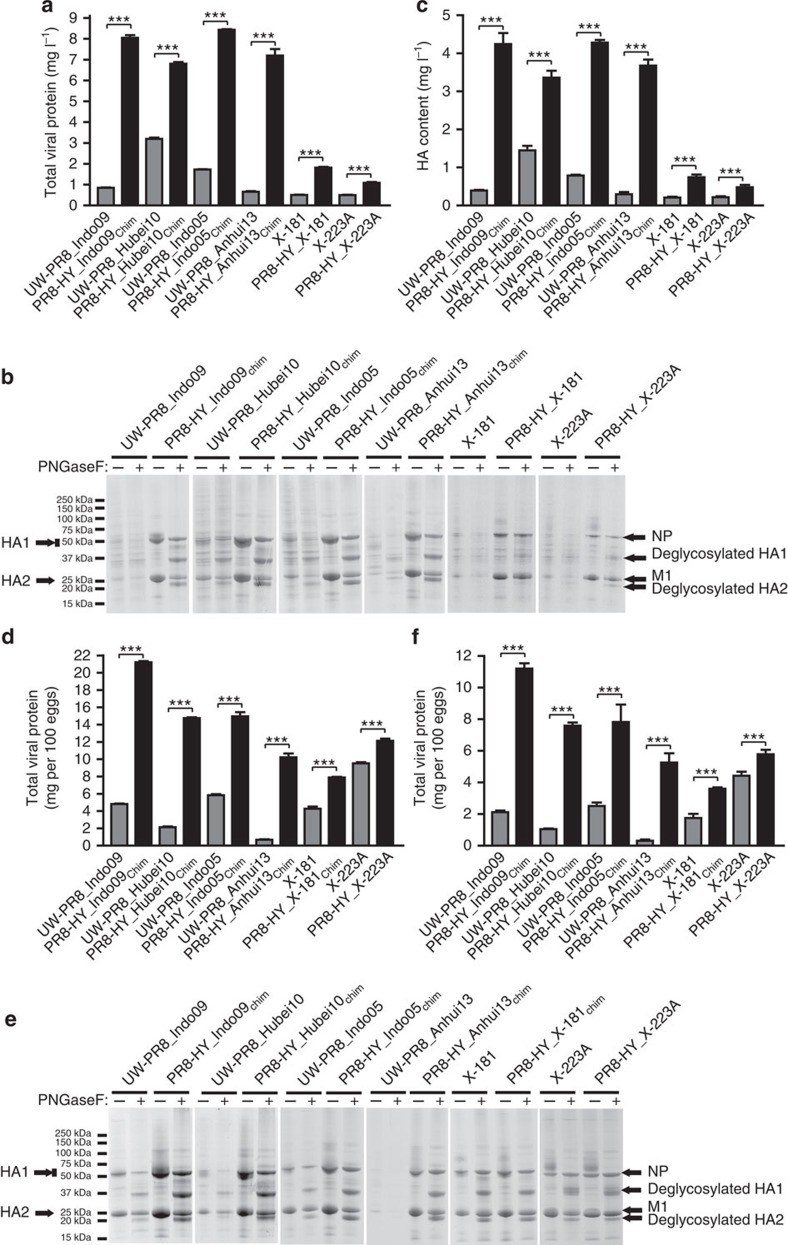
Evaluation of the total viral protein and HA content of PR8-HY candidate vaccine viruses. Total viral protein yield of Vero cell- (**a**) or egg-grown (**d**), sucrose gradient-purified virus samples. The total protein content of virus concentrates was measured by using the Pierce BCA assay kit (Thermo Fisher) according to the manufacturer's instructions. SDS–PAGE analyses of virus samples from Vero cells (**b**) or embryonated chicken eggs (**e**). Virus concentrates were deglycosylated with PNGase (PNFase F+) or left untreated (PNGase F−). HA contents of Vero-; (**c**) and egg-grown (**f**) viruses. The HA contents were calculated based on the total viral protein amounts (Fig. 7a,d) and the relative amounts of HA (Fig. 7b,e); for details, see Study Design. The HA contents are expressed in mg l^−1^ (for Vero cell-grown viruses) or mg per 100 eggs (for viruses grown in embryonated chicken eggs). Asterisks indicate a significant difference. The values presented are the average of three independent experiments±s.d. *P*-values were calculated by using Tukey's *post-hoc* test, comparing the total viral protein yield and HA content of wild-type viruses with that of recombinant high-yield vaccine viruses; ***P<0.005.

Inactivated influenza vaccines are standardized by HA content; the most commonly used vaccine preparations contain 15 μg each of H1 HA, H3 HA and type B HA. Total viral protein yield and HA content are therefore important parameters in vaccine optimization. Here, we compared the total viral protein yield and HA content of our selected vaccine candidates propagated in Vero cells or embryonated chicken eggs. Viruses collected from cell culture supernatants or egg allantoic fluid were concentrated and purified by use of sucrose gradient centrifugation. Total viral protein yield was determined using the Pierce BCA Protein Assay Kit (Thermo Scientific, Rochester, NY, USA). In comparison with the UW-PR8-based control viruses, the PR8-HY backbone yielded significantly higher amounts of total viral protein (Fig. 7a,d). To evaluate the HA content, the purified virus samples were deglycosylated with PNGase F (thus allowing the detection of HA2, which in its glycosylated form is similar in size to M1) and then analysed by using SDS–polyacrylamide gel electrophoresis (SDS–PAGE) (Fig. 7b,e and Supplementary Fig. 4). Based on a densitometry analysis, the amounts of HA1, HA2, NP and M1 were determined. To calculate the HA content, we divided the HA amount (calculated by summing the amounts of HA1 and HA2) by the sum of the amounts of HA1, HA2, NP and M1, and multiplied this value by the amount of total viral protein in the samples analysed via gel electrophoresis. The results showed that PR8-HY-based vaccine candidates displayed a significantly higher HA content than those possessing the PR8-UW backbone (Fig. 7c,f). We have not yet performed single radial immunodiffusion assays or assessed the ratio of infectious to physical particles.

### Virulence of PR8-HY-based vaccine viruses in mice

To rule out the possibility that the high-yield-conferring mutations in the PR8-HY backbone render the virus highly virulent in mammals, we compared the virulence in mice of UW-PR8_Indo05, PR8-HY_Indo05 and PR8-HY_Indo05_Chim_. The doses required to kill 50% of infected animals (mouse lethal dose 50, MLD_50_) were 10^2.5^, 10^2^ and 10^2.25^ PFU, respectively (Supplementary Fig. 5a). Mice infected with PR8-HY-backbone-based viruses also lost more weight (Supplementary Fig. 5b) and had higher lung virus titres on days 3 and 5 post infection than did mice infected with the UW-PR8-based virus (Supplementary Fig. 5c), suggesting that the higher replicative ability of PR8-HY-based viruses may cause slightly higher mouse virulence. However, these increases in virulence were small and PR8-HY-based vaccines would be administered as inactivated vaccines; therefore, the slightly higher mouse virulence of the PR8-HY backbone compared with the UW-PR8 backbone should not be a major issue for vaccine production, although additional studies may be needed in the future to address this issue.

### Genetic stability of the PR8-HY vaccine backbone

Vaccine viruses need to have high genetic stability to minimize the risk of emergence of variants with unwanted properties. To evaluate the genetic stability of our novel vaccine candidate, PR8-HY_Indo05 and PR8-HY_Indo05_Chim_ were each passaged 10 times in Vero cells and embryonated chicken eggs, and their whole genome sequences were then analysed. None of the yield-enhancing mutations converted to wild-type sequence during passages in cultured cells or embryonated chicken eggs, and no additional mutations were detected in any of the viral genes. In addition, we inoculated the viruses shown in Table 1 into MDCK cells (at an MOI of 0.001), Vero cells (at an MOI of 0.005) or embryonated chicken eggs (2 × 10^3^ PFU per egg). Forty-eight hours later, we collected cell culture supernatants and allantoic fluids and sequenced the HA and NA genes of the amplified viruses; no mutations were found in these genes. These findings further support the vaccine potential of the PR8-HY backbone.

## Discussion

Human infections with avian H5N1 or H7N9 viruses have raised concerns about a looming pandemic and the ability of standard influenza vaccine production methods to supply sufficient amounts of an effective vaccine in a limited time. Vaccine viruses that replicate to higher titres than current vaccine viruses would lessen these concerns and also avoid production delays with seasonal vaccines in the event that the original vaccine virus candidates grow to low titres. Through a comprehensive study that combined random and site-directed mutagenesis of different functional regions of the viral genome (that is, coding, non-coding and promoter regions), we generated a high-yield PR8-based vaccine backbone that significantly increased the yield of pandemic H5N1 and H7N9 vaccine candidates, and of seasonal H1N1 and H3N2 vaccine viruses. Although this vaccine backbone was selected for its high-yield properties in Vero and MDCK cells, it also improved vaccine virus yields in embryonated chicken eggs.

Vaccine production in cell culture has a number of possible benefits over production in embryonated eggs. The biggest drawback of influenza virus amplification in embryonated chicken eggs is the emergence of antigenic variants^4^,^5^,^6^,^7^,^8^,^9^,^10^,^11^. Such unwanted adaptation may have contributed to the reduced efficacy of some influenza vaccines^12^,^13^. In contrast, the HAs of cell culture-propagated viruses are typically identical to those of the original isolates^4^,^5^,^6^,^7^,^12^,^36^,^37^,^38^,^39^,^40^,^41^,^42^,^43^,^44^,^45^. Moreover, influenza viruses isolated from humans often replicate more efficiently in cultured mammalian cells than in embryonated chicken eggs^46^,^47^. Several studies have also suggested that human influenza vaccines prepared from viruses propagated in cultured cells induce better protective immunity than those grown in embryonated chicken eggs^9^,^48^,^49^,^50^,^51^,^52^.

Despite the advantages of cell culture-based influenza vaccines compared with egg-grown products, the former represent only a small percentage of the influenza vaccine market. Vero cells have been used for the production of inactivated poliovirus^53^, rabies^54^ and live smallpox vaccines^55^, resulting in an extensive track record for Vero cell-derived vaccines for human use. In October 2009, a Vero cell-based vaccine to the novel pandemic H1N1 virus was developed and licensed in Europe (http://www.baxter.com/press_room/press_releases/2009/10_07_09-celvapan.html). One MDCK cell-derived vaccine has been available in Europe since 2001 (Influvac, Solvay Pharmaceuticals) and a second since 2007 (Optaflu, Novartis), but such vaccines were not available in the United States until 2012 (Flucelvax, Novartis) (http://www.fda.gov/newsevents/newsroom/pressannouncements/ucm328982.htm).

Our high-yield vaccine backbone, PR8-HY, in some instances in combination with chimeric HA and NA viral RNA segments, increased peak virus titres in Vero cells 4.6−269-fold compared with PR8-UW-based vaccines (Table 1). In MDCK cells and embryonated chicken eggs, virus titres increased 1.4−29-fold and 4.6–172-fold, respectively (Table 1). The increases in titre conferred by the PR8-HY backbone for pandemic H5N1 and H7N9 vaccine candidates were substantial; it is not yet clear why smaller improvements in titre were found for seasonal human vaccine viruses in MDCK and Vero cells. It should be kept in mind that most comparisons in Table 1 were made against the control strain UW-PR8, which replicates to higher titres than other PR8 strains^28^,^29^.

The high-yield vaccine backbone PR8-HY carries seven amino-acid changes across five different viral proteins compared with the UW-PR8 strain: PB2-I504V, PB1-M40L/G180W, PA-R401K, NP-I116L and NS1-A30P/R118K. A number of growth-enhancing mutations described in the literature did not increase the replicative ability of UW-PR8, most likely because many mutations in influenza viral proteins have context-specific effects (that is, their effect depends on the virus used for testing). The context-specific effects of many mutations, and the fact that random mutagenesis may create mutants that do not frequently emerge in nature (for example, those that require two mutations in one codon) most likely also explain why the PB2-I504V mutation was the only amino-acid change identified both through screens of random libraries and through the testing of potentially yield-enhancing mutations previously described in the literature. Interestingly, Rolling *et al*.^56^ detected this mutation in three independent studies of adaptation of PR8 virus to Mx1-expressing mice, suggesting that PB2-V504 increases the replicative ability of PR8 virus. Consistent with this hypothesis, database searches revealed that >99% of all human and avian PB2 proteins encode valine at position 504 (Supplementary Table 13); in fact, we found only 39 human and 4 avian viruses that encode PB2-I504. Hence, our high-yield vaccine virus acquired the amino acid commonly found at this position. The biological significance of the PB2-I504V mutation, which is located at the surface of PB2 (ref. 57), is yet not known.

The significance of the PB1-M40L and PB1-G180W mutations is also currently not known. At position 40, methionine is highly conserved among human and avian PB1 proteins. By contrast, at position 180 of PB1, >99% of human and avian PB1 proteins encode glutamic acid, whereas glycine (as encoded by PR8 viruses) is found very rarely (Supplementary Table 13). Interestingly, an analysis of the PB1 proteins of recent seasonal influenza vaccines found a PB1-G180E mutation in 11 of 21 vaccine viruses^58^. Tryptophan (as found in our study) may have been selected from our mutant virus library because its length is similar to that of glutamic acid, although these amino acids differ in ‘bulkiness', hydrophobicity and charge.

The PA-R401 and NP-I116 residues are highly conserved among human and avian influenza A virus proteins, while the lysine and leucine residues found at these positions of the PR8-HY backbone are very rare (Supplementary Table 13). No biological functions have been identified for these residues. Likewise, no functions have been identified for the amino acids at positions 30 and 118 of NS1. The NS1-A30P mutation detected in PR8-HY has not been observed in nature (Supplementary Table 13). The NS1-K118 residue encoded by the PR8-HY backbone is expressed by roughly one-third of avian NS1 proteins, while most avian and almost all human NS1 proteins possess arginine at this position (Supplementary Table 13).

The influenza virus promoter sequences at the 5′ and 3′ ends of the negative-sense viral RNA and the positive-sense complementary RNA are highly conserved, with the exception of the fourth positions from the 3′ end of the viral RNA. At this position, the polymerase genes have been reported to encode a C residue, whereas the remaining five RNA segment encode a U residue. Consistent with the earlier studies^35^, we found that C4U replacement increased viral replicative ability. By contrast, modifications at other positions of the promoter region of the HA segment did not confer growth advantages.

Several studies have found that chimeric HA and NA proteins (in which the intracellular and transmembrane domains are derived from PR8 virus, while the extracellular domains are derived from a (candidate) vaccine virus) confer a growth advantage over viruses that possess the full-length HA and NA genes/proteins of the respective vaccine virus^22^,^23^,^24^,^25^,^26^,^27^. The underlying mechanism is not fully understood but may reflect incompatibilities among the proteins and/or viral packaging signals of the viral components derived from the different viruses. Incompatibility among viral proteins and/or packaging signals may also explain why we did not detect a consistent pattern; while chimeric HA and NA segments provided clear advantages with some vaccine candidates (for example, A/Hubei/1/2010 and A/Anhui/1/2013), no significant differences were found for other candidates. In the event of a pandemic, both versions of candidate vaccines could be generated and compared. Nowadays, this can be achieved conveniently through the use of chemically synthesized HA and NA genes.

In conclusion, we here present a high-yield PR8 vaccine virus backbone that could improve the titres of pandemic and seasonal influenza vaccines in both cultured cells and embryonated chicken eggs.

## Methods

### Study design

Our studies were designed to develop influenza vaccine viruses with increased yield in mammalian cells and/or embryonated chicken eggs. The details of the study design are described below.

### Viruses and cells

293T human embryonic kidney cells (obtained from American Type Culture Collection (ATCC)) were maintained in DMEM supplemented with 10% fetal bovine serum. MDCK cells (obtained from ATCC) were grown in MEM containing 5% new born calf serum. Vero cells (obtained from ATCC) were grown in MEM containing 10% fetal bovine serum. Vaccine virus NYMC X-181 (derived from A/California/07/2009 (pandemic H1N1)) and NYMC X-223A (derived from A/Texas/50/2012 (seasonal H3N2)) were kindly provided by the National Institute for Biological Standards and Control (NIBSC; Potters Bar, UK). All other viruses were generated by use of reverse genetics.

### Construction of plasmids

The detoxified HA and NA for VN04 were generated previously^59^. The wild-type sequences for the HA and NA genes of A/Hubei/1/2010 (H5N1), A/Egypt/N03072/2010 (H5N1), A/Indonesia/05/2005 (H5N1) and A/Anhui/1/2013 (H7N9) were obtained from GISAID.org (Supplementary Table 14). The H5N1 gene segments (NA and detoxified HA) were engineered by site-directed mutagenesis of related sequences or were chemically synthesized (Genescript, USA), then amplified by PCR and inserted into the RNA polymerase I vector pHH21 (ref. 60). The Anhui13 HA and NA sequences were chemically synthesized and cloned into pHH21 as above. The HA and NA genes of X-181 and X-223A were amplified by PCR with reverse transcription (RT–PCR) from the respective vaccine virus and cloned into the pHH21 vector.

To rapidly construct chimeric HA and NA segments, universal chimeric HA and NA vectors were constructed. Briefly, the pHH21-PR8-HA and pHH21-PR8-NA plasmids were modified to contain two BsmBI restriction enzyme sites that were then used to replace the HA and NA ecto-domain sequences, respectively. This cloning strategy does not introduce unwanted amino-acid changes into the HA and NA sequences.

### Construction of plasmid libraries

Random mutations were introduced into the six internal genes of UW-PR8 virus by error-prone PCR using GeneMorph II Random Mutagenesis Kit. Briefly, PCR reaction conditions and target DNA template amounts were optimized to generate 1–4 amino acids substitutions per protein. The randomly mutated cDNAs were then inserted into RNA polymerase I vector^60^ to generate randomly mutated plasmid libraries. The diversity of the plasmid libraries was confirmed by sequence analysis: we found that 75–92% of the sequenced clones retained the start codon, lacked premature stop codons, and possessed, on average, 1.95–3.9 amino acid changes per PCR product.

### Virus rescue and virus library generation

All viruses and virus libraries used in this study were generated by means of reverse genetics, using eight pHH21-based RNA polymerase I plasmids for viral RNA synthesis and four protein-expressing plasmids to synthesize the viral replication complex as described by Neumann *et al*.^60^ Virus stocks were generated by infecting MDCK cells, Vero cells or embryonated chicken eggs with 100 μl of virus-containing supernatant derived from plasmid-transfected 293T cells. The titres of all virus stocks were determined by means of plaque assays in MDCK cells. To generate virus libraries, cells were transfected with a mutant plasmid library instead of the wild-type construct. At 48 h post transfection, supernatants derived from plasmid-transfected 293T cells were passaged in MDCK and Vero cells by infecting them at a MOI of 0.01. All experiments involving wild type or reassortant A/Anhui/1/2013 (H7N9) virus were carried out in biosafety level 3 containment. Experiments with H5N1 viruses that were exempt from Select Agent status by APHIS and approved by the University of Wisconsin-Madison Institutional Biosafety Committee for work at BSL-2 were used at that containment level.

### Evaluation of viral growth kinetics

To analyse the growth characteristics of viruses, Vero or MDCK cells were infected in triplicate (a sample size adequate to detect large effects between groups) with recombinant viruses at a MOI of 0.001 (MDCK cell infection) or 0.005 (Vero cell infection). One hour after incubation at 37 °C, the cells were washed once with phosphate-buffered saline (PBS), and fresh MEM/BSA medium with 0.5 μg ml^−1^ tosyl phenylalanyl chloromethyl ketone (TPCK) trypsin was added. Supernatants were collected at the indicated time points and the virus titres in the supernatants were determined by plaque assays in MDCK cells. For Vero cell infections, fresh TPCK trypsin was added to the supernatants every day (0.5 μg ml^−1^). To analyse viral growth kinetics in embryonated chicken eggs, 2 × 10^3^ PFU of virus was inoculated into 10-day-old embryonated chicken eggs. The allantoic fluids of four eggs each were harvested at the indicated time points. Virus titres were determined by plaque assays in MDCK cells.

The haemagglutination (HA) titres of supernatants derived from infected MDCK and Vero cells or allantoic fluid derived from inoculated eggs were determined by using an HA assay. Briefly, 50 μl of virus sample was serially diluted 2-fold in 96-well U-bottom microtitre plates (Thermo Scientific) that contained 50 μl of PBS per well, and then 50 μl of 0.5% turkey red blood cells was added to each well. After a 45-min incubation at room temperature, the highest agglutinating well was read as the virus HA titre.

### Virus concentration and purification

Two 4-Layers Easy-Fill Cell Factories (Thermo Scientific) of Vero cells were infected with recombinant viruses, and fresh TPCK trypsin was added every day (0.5 μg ml^−1^). Alternatively, viruses were grown in 10-day-old embryonated chicken eggs. Infected cell culture supernatants were collected at 72 h post infection, whereas allantoic fluids were collected 48 h after egg inoculation. Cell culture supernatants or allantoic fluids were clarified by centrifugation (3,500 r.p.m., 15 min, 4 °C). Viruses were then pelleted by centrifugation (18,500 r.p.m., 90 min at 4 °C in a Beckman Type19 rotor), resuspended in 5 ml of PBS and loaded onto 30 ml, 20–50% continuous sucrose gradients that were centrifuged at 25,000 r.p.m. for 90 min at 4 °C in a Beckman SW32 rotor. The virus band was collected, diluted in PBS, pelleted by centrifugation (25,000 r.p.m., 90 min, 4 °C in a Beckman SW32 rotor), and the final virus pellet was resuspended in 400 μl of PBS (including 0.1% β-propiolactone (BPL)) overnight at 4 °C to inactivate the virus particles. Then, samples were incubated at 37 °C for 45 min to inactivate the BPL, aliquoted, and stored at −80 °C.

A/Anhui/1/2013 (H7N9) recombinant viruses were amplified in BSL-3 containment and inactivated by treatment with 0.1% BPL overnight. Virus inactivation was confirmed by negative HA assays after two consecutive passages in embryonated chicken eggs (the method and validation of virus inactivation were approved by the Select Agent Program of the University of Wisconsin-Madison).

### Total protein assay

Total protein content of virus concentrates was determined by using the Pierce BCA protein assay kit (Thermo Scientific) according to the manufacturer's instructions.

### Deglycosylation of viral proteins using PNGase F

Virus proteins were deglycosylated by using PNGase F (New England Biolabs). Specifically, 10 μl of virus concentrates were denatured according to the manufacturer's instructions in a total reaction volume of 60 μl, followed by incubation at 37 °C for 20 h with 2 μl of a 1/10 dilution of PNGase F enzyme in the buffer provided by the manufacturer and with NP40 at a final concentration of 1%.

### SDS–PAGE electrophoresis

One microlitre of virus concentrate was mixed with water to a total volume of 10 μl. Loading dye (2.5 μl) with 2% (v/v) β-mercaptoethanol as the reducing agent was added to each sample. Samples were heated to 95 °C for 5 min before loading onto the NuPage 4–12% Bris-Tris precast gel (Life technology). Gels were run at 150 V for 120 min using 1 × MES buffer (Bio-Rad) and then stained with SYPRO-Ruby (Sigma). Quantification of protein amounts was carried out by using ImageJ software (National Institutes of Health). To calculate the HA content, we divided the HA amount (calculated by summing the amounts of HA1 and HA2) by the sum of the amounts of HA1, HA2, NP and M1, and multiplied this value by the amount of total viral protein in the samples analysed by use of gel electrophoresis.

### Virulence studies in mice

To determine the MLD_50_, three mice/group (a sample size adequate to detect large effects between groups) of 6-week-old female BALB/c mice (Jackson Laboratory, Bar Harbor, ME, USA) were anaesthetized with isoflurane and inoculated intranasally with 10-fold dilutions of virus (from 10^1^ to 10^6^ PFU) in a volume of 50 μl. For these experiments, mice were randomized and investigators were not blinded. Body weight changes and survival were recorded daily until day 14, and the MLD_50_ was calculated by the method of Reed and Muench. To assess virus replication in mice, 10^4^ PFU of recombinant viruses were used to infect six additional mice. At days 3 and 5 post infection, three mice in each group were euthanized and their lungs were collected and homogenized. Virus titres were determined by plaque assays in MDCK cells.

### Genetic stability testing

To evaluate the genetic stability of the high-yield backbone, the recombinant viruses were consecutively passaged 10 times in Vero cells at an MOI of 0.1. In parallel, these viruses were also consecutively passaged 10 times in embryonated chicken eggs at an inoculation dosage of 2 × 10^4^ PFU per egg. Viruses sampled after the 10th passage in the supernatants of Vero cells and the allantoic fluid of embryonated chicken eggs were sequenced by means of Sanger sequencing.

### Virus genome sequencing

To determine the mutations present in the 36 high-yield candidate viruses isolated from the MDCK passages, we performed whole genome sequencing on the Ion Torrent PGM System (Life Technologies). Briefly, 5 μl of total RNA extracted from the supernatant of virus-infected cells was used to amplify all eight viral segments in a multi-segment RT–PCR reaction^61^. Barcoded, 200-base insert libraries were then produced using the Ion Xpress Plus Fragment Library Kit (Life Technologies), with an eight-cycle limited PCR to increase the numbers of fragments with adaptors. Final library pools were constructed containing equimolar amounts of each barcoded component, and the pools were run on Ion Torrent 314 chips.

### Statistical analysis

The data were analysed by using the R software (www.r-project.org), version 3.1. For comparisons of multiple groups with measurements collected independently at different time points (that is, viral growth curves in embryonated chicken eggs), we used two-way analysis of variance followed by Tukey's *post-hoc* test. For comparisons of measurements from multiple groups collected at a single time point, we used one-way analysis of variance also followed by Tukey's *post-hoc* test. For comparisons of multiple groups with dependent measurements (that is, viral growth curves in cell culture for which aliquots were collected from the same culture at different time points), we fitted a linear mixed-effects model to the data using the R package NLME, and the time, the virus strains, and the interaction between these two factors were considered. Next we built a contrast matrix to compare the strains in a pairwise fashion at the same time points (for example, group_1 versus group_2 at 24 h post infection, group_1 versus group_3 at 24 h post infection, group_2 versus group_3 at 24 h post infection), using the R package PHIA. Because the comparisons were performed individually, the final *P*-values were adjusted using Holm's method to account for multiple comparisons. Finally, for comparisons involving only two groups with measurements at single time points, we used two-tailed, unpaired *t*-tests; if multiple comparisons were performed, the *P*-values were adjusted using Holm's method.

In all cases, except for the data shown in Fig. 7, raw data were converted to the logarithmical scale before the analysis and the results were considered statistically significant if we obtained *P*-values (or adjusted *P*-values)<0.05, and the variance between groups was assessed using Levene's test (it was similar for the groups being compared, with *P*-value>0.05).

### Ethics and biosafety

Our experiments in mice followed the University of Wisconsin-Madison's Animal Care and Use Protocol. All experiments were approved by the Animal Care and Use Committee of the University of Wisconsin-Madison (protocol number V00806), which acknowledged and accepted both the legal and ethical responsibility for the animals, as specified in the Fundamental Guidelines for Proper Conduct of Animal Experiment and Related Activities in the Animal Welfare Act and associated Animal Welfare Regulations and Public Health Service Policy (USA).

All experiments were completed before the US Government announced a research pause on certain gain-of-function studies on 17 October 2014.

## Additional information

**How to cite this article:** Ping, J. *et al*. Development of high-yield influenza A virus vaccine viruses. *Nat. Commun*. 6:8148 doi: 10.1038/ncomms9148 (2015).

## Supplementary Material

Supplementary Information Supplementary Figures 1-5, Supplementary Tables 1-14, Supplementary References

## Figures and Tables

**Table 1:** Comparison of growth characteristics of PR8-HY viruses possessing wild-type or chimeric HA and NA genes.

Virus	Growthsubstrate	Peak virus titre			Peak HA titre			Total viral protein yield		HA content	
		PFU ml^−1^	Statistical significance compared with UW-PR8	Fold-increase versusUW-PR8	2^n^	Statistical significance compared with UW-PR8	Fold-increase versusUW-PR8	Statistical significance compared with UW-PR8	Fold-increase versusUW-PR8	Statistical significance compared with UW-PR8	Fold-increase versusUW-PR8
PR8-HY_Indo09_Chim_(H5N1)	Vero	1.0 × 10^9^	*P*<0.005	28.5	9.5–10	*P*<0.005	5.7	*P*<0.005	9.8	*P*<0.005	10.8
	MDCK	1.0 × 10^9^	*P*<0.005	4.6	9.5	*P*<0.005	3	ND*	ND	ND	ND
	Egg	7.0 × 10^9^	*P*<0.005	5.9	11–11.5	*P*<0.005	2.9	*P*<0.005	4.4	*P*<0.005	5.3
PR8-HY_VN04_Chim_(H5N1)	Vero	8.8 × 10^8^	*P*<0.005	5.1	8.5	*P*<0.005	2.2	*P*<0.005	1.8	*P*<0.005	1.9
	MDCK	5.8 × 10^8^	*P*<0.01	1.4	9	*P*<0.005	1.3	ND	ND	ND	ND
	Egg	2.7 × 10^9^	*P*<0.005	4.6	10	—†	2	ND	ND	ND	ND
PR8-HY_Hubei10_Chim_(H5N1)	Vero	7.6 × 10^8^	*P*<0.005	9.5	8.5–9	*P*<0.005	3.5	*P*<0.005	2.1	*P*<0.005	2.3
	MDCK	2.2 × 10^8^	*P*<0.01	1.8	8.5	*P*<0.005	1.3	ND	ND	ND	ND
	Egg	4.0 × 10^9^	*P*<0.005	29	9.5–10	*P*<0.005	4.9	*P*<0.005	6.9	*P*<0.005	7.3
PR8-HY_Egypt10_Chim_(H5N1)	Vero	1.3 × 10^9^	*P*<0.005	221	10.5–11	*P*<0.005	76	*P*<0.005	9.7	*P*<0.005	11.3
	MDCK	6.1 × 10^8^	*P*<0.005	3.6	9–9.5	*P*<0.005	1.7	ND	ND	ND	ND
	Egg	4.7 × 10^9^	*P*<0.005	7.5	12.5	—	2.7	ND	ND	ND	ND
PR8-HY_Indo05_Chim_(H5N1)	Vero	8.5 × 10^8^	*P*<0.005	28.6	9	*P*<0.005	4.5	*P*<0.005	4.9	*P*<0.005	5.5
	MDCK	7.1 × 10^8^	*P*<0.005	2.4	9.5	*P*<0.005	3	ND	ND	ND	ND
	Egg	7.3 × 10^9^	*P*<0.005	12.5	12.5–13	*P*<0.005	4.6	*P*<0.005	2.6	*P*<0.005	3.1
PR8-HY_Anhui13_Chim_(H7N9)	Vero	1.3 × 10^9^	*P*<0.005	173	8–8.5	*P*<0.005	3.6	*P*<0.005	9.9	*P*<0.005	12.4
	MDCK	1.5 × 10^9^	*P*<0.005	29	9.5–10	*P*<0.005	5.5	ND	ND	ND	ND
	Egg	3.6 × 10^9^	*P*<0.005	172	11.5	*P*<0.005	17.7	*P*<0.005	14.8	*P*<0.005	16.9
PR8-HY_X-181_Chim_ orPR8-HY_X-181‡(H1N1)	Vero	1.3 × 10^8^	*P*<0.005§	269§	8	*P*<0.005§	134§	*P*<0.005§	3.3§	*P*<0.005	3.9§
	MDCK	3.3 × 10^8^	*P*<0.005§	4.7§	9.5	—	1.6§	ND	ND	ND	ND
	Egg	7.2 × 10^9^	*P*<0.005§	5§	11.5–12	*P*<0.05§	2.7§	*P*<0.005§	1.8§	*P*<0.005	2.1§
PR8-HY_X-223A(H3N2)	Vero	3.7 × 10^8^	*P*<0.005§	9.3§	9–9.5	*P*<0.005§	2.3§	*P*<0.005§	2.1§	*P*<0.005	2.2§
	MDCK	6.7 × 10^8^	*P*<0.005§	5.3§	9.5	*P*<0.05§	2§	ND	ND	ND	ND
	Egg	3.8 × 10^9^	*P*<0.05§	6§	11–11.5	—	1.3§	*P*<0.005§	1.3§	*P*<0.005	1.3§

^*^ND: not determined.

^†^No significant difference.

^‡^Since PR8-HY_X-181_Chim_ does not grow in Vero cells, PR8-HY_X-181 was used to evaluate total viral protein and HA content in Vero cells.

^§^Comparisons were carried out between high-growth X-181 and wild-type X-181 viruses, or between high-growth and wild-type X-223A viruses, respectively.
