# Supplementary material for: Development of high-yield influenza A virus vaccine viruses
Source: Nat Commun. 2015 Sep 2;6:8148. doi: 10.1038/ncomms9148 (PMC4569720; doi:10.1038/ncomms9148)
Supplement: Supplementary Information — Supplementary Figures 1-5, Supplementary Tables 1-14, Supplementary References [file ncomms9148-s1.pdf]

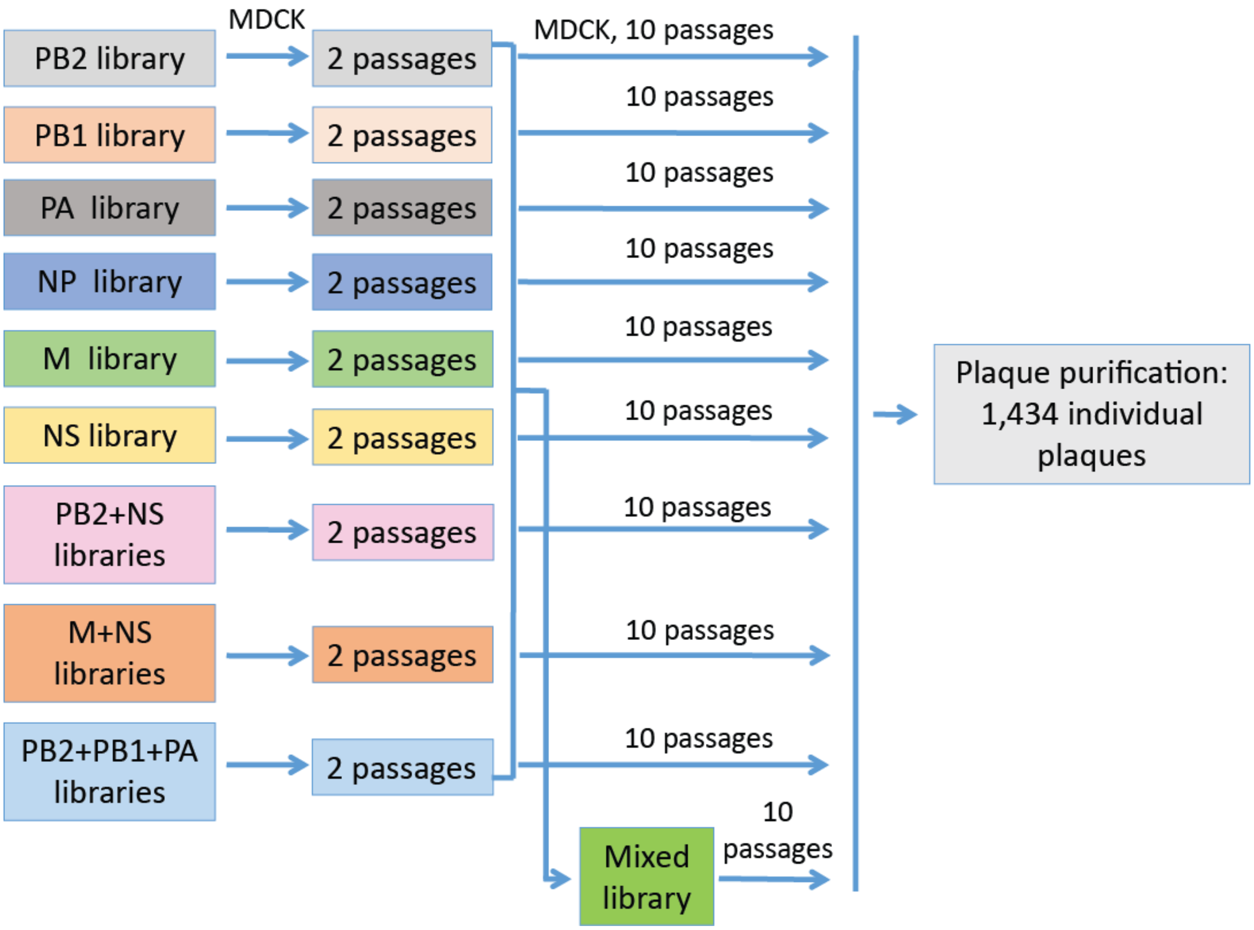

Supplementary Figure 1 Schematic overview of the mutant virus libraries and their consecutive passages in MDCK cells. All nine virus libraries were passaged twice in MDCK cells. Then, aliquots of all nine virus libraries were combined, resulting in a 'mixed library'. The mixed and individual libraries were passaged ten more times in MDCK cells, followed by the isolation of 1,434 individual plaques.

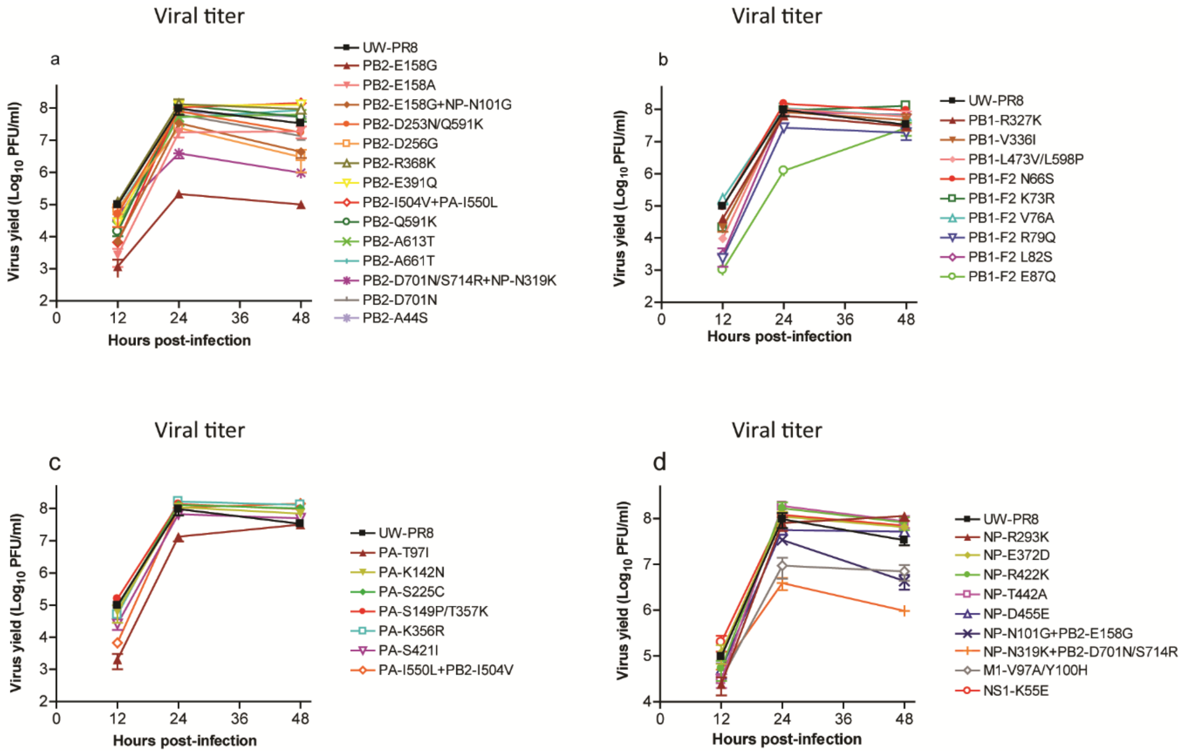

Supplementary Figure 2 Growth kinetics in MDCK cells of UW-PR8 mutants possessing mutations identified in the literature. Amino acid changes in viral proteins that may affect UW-PR8 replication kinetics (see Supplementary table 2) were introduced into UW-PR8 and tested for their replication kinetics in MDCK cells. The mutations tested were in the PB2 (**a**), PB1 or PB1-F2 (**b**), PA (**c**), or NP, M1, or NS1 (**d**) proteins. When two amino acid changes in two different proteins were tested together, the respective changes are listed in both panels.

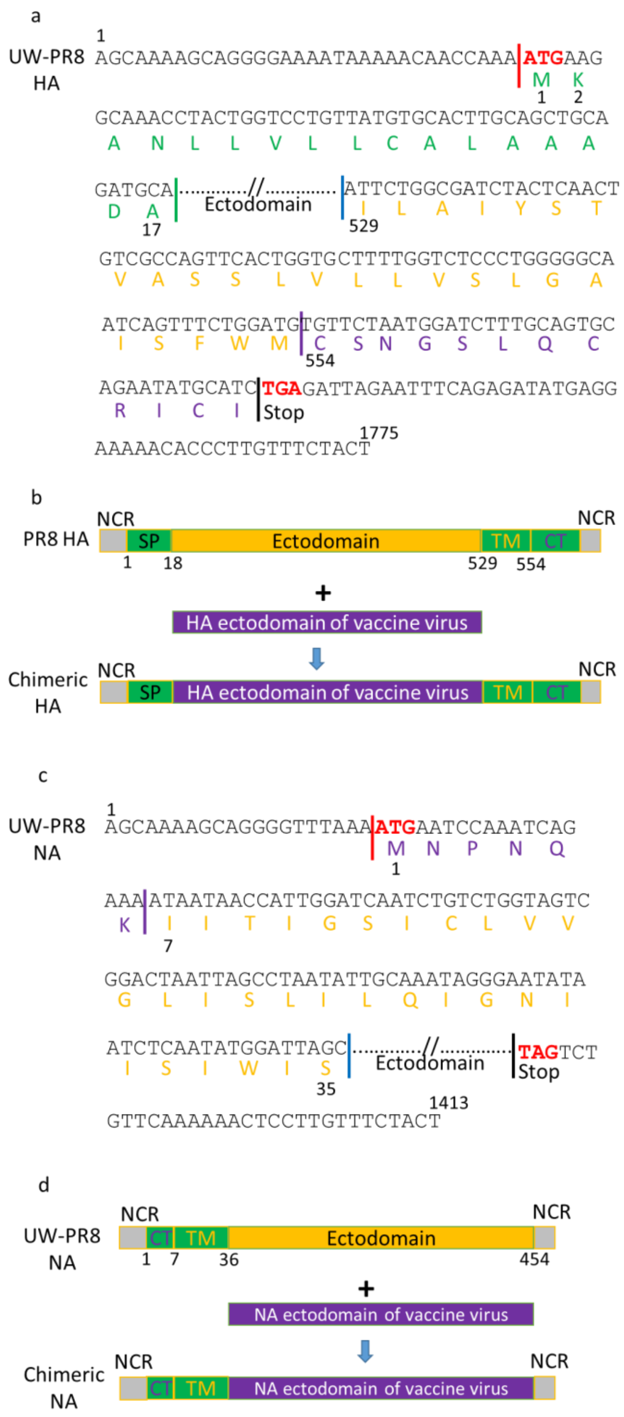

Supplementary Figure 3 Schematic diagram of chimeric HA and NA genes. (**a**) Sequence of the UW-PR8 HA gene. Shown are the sequences of the signal peptide (green), transmembrane domain (yellow), and cytoplasmic tail (purple). The start and stop codons of HA are indicated in red. The ectodomain of UW-PR8 HA was replaced with that of the respective vaccine virus. Numbers above nucleotides refer to nucleotide positions. Numbers below amino acids refer to amino acid positions. (**b**) Schematic diagram of chimeric HA constructs. NCR, noncoding region. Numbers refer to amino acid positions (**c**) Sequence of the UW-PR8 NA gene. Shown are the cytoplasmic tail (purple) and transmembrane domain (yellow). The start and stop codons of NA are indicated in red. The ectodomain of UW-PR8 NA was replaced with that of the respective vaccine virus. Numbers above nucleotides refer to nucleotide positions. Numbers below amino acids refer to amino acid positions. (**d**) Schematic diagram of chimeric NA constructs. NCR, noncoding region. Numbers refer to amino acid positions.

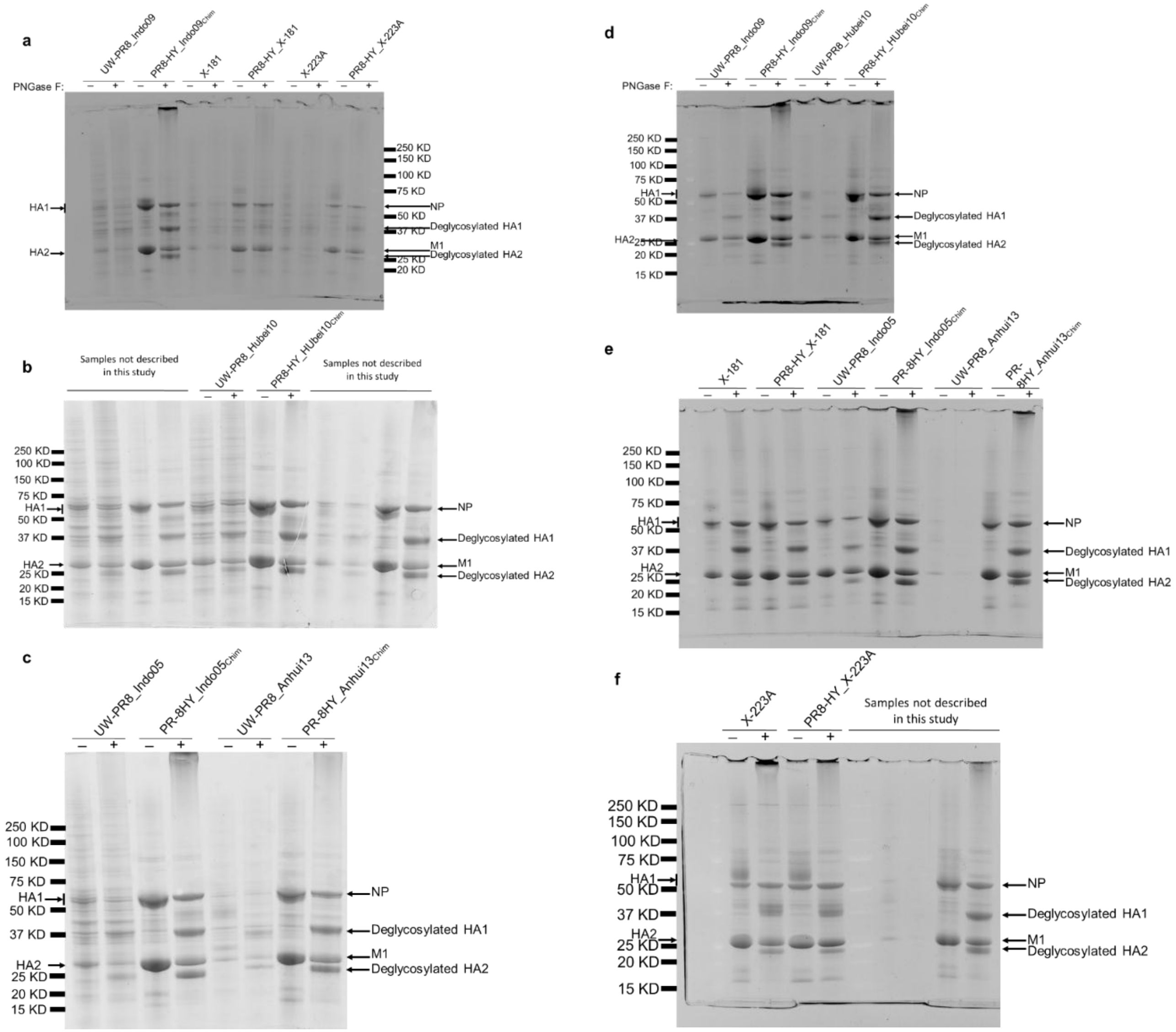

Supplementary Figure 4 SDS-PAGE analysis of concentrated, purified viruses. Vero cell-grown (a, b and c) or egg-grown viruses (d, e and f) were deglycosylated with PNGase (PNFase F+) or left untreated (PNGase F-).

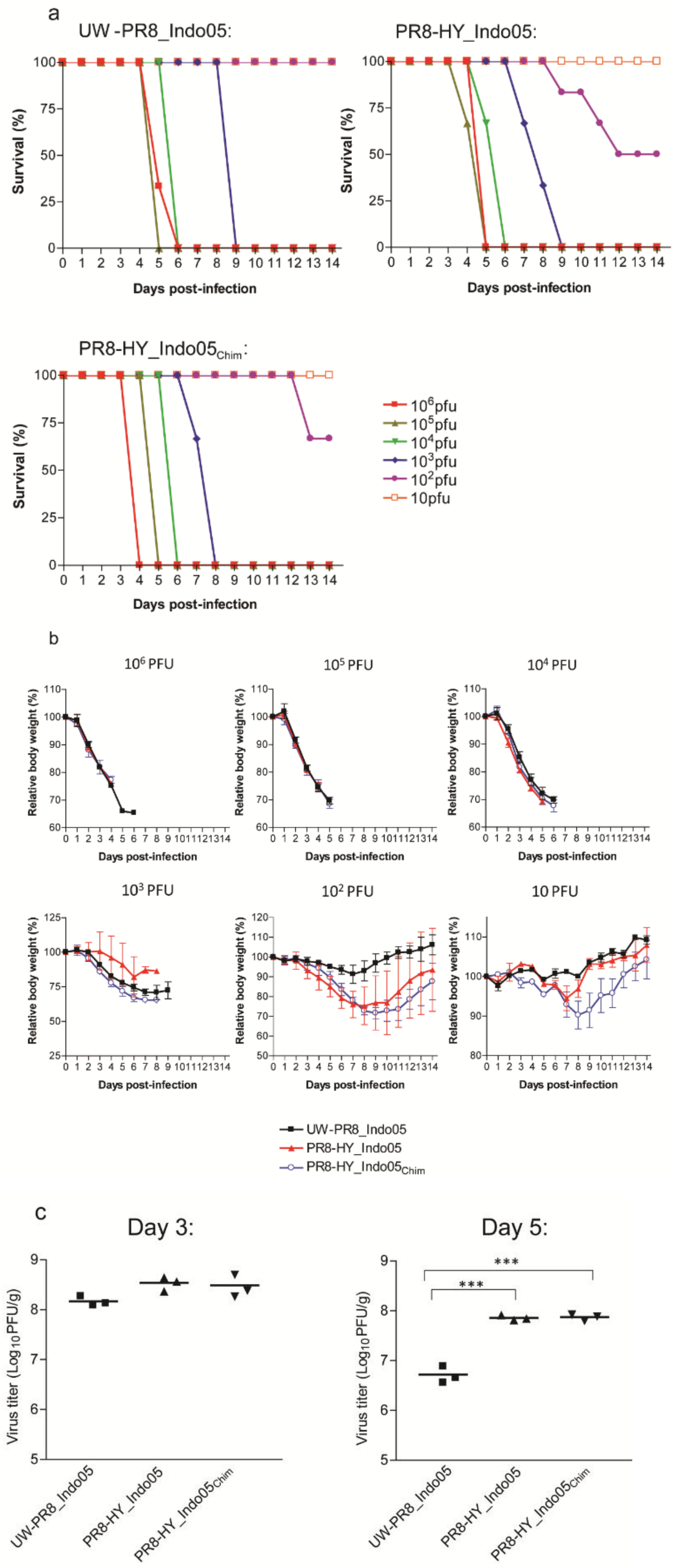

Supplementary Figure 5 Virulence of PR8-HY viruses in mice. (**a**) To determine the MLD_{50} values of the PR8-HY viruses, BALB/c mice were inoculated intranasally with the indicated doses of UW-PR8_Indo05, PR8-HY_Indo05, or PR8-HY_Indo05$_{Chim}$ viruses. Survival (**a**) and body weight changes (**b**) were recorded daily until day 14. To determine virus replication in mice, 10^4 PFU of each virus was used to infect six additional mice. At days 3 and 5 post-infection, three mice in each group were euthanized and lung virus titers were determined by plaque assays in MDCK cells (**c**).

Supplementary Table 1. Mutations identified in high-yield viruses isolated from virus libraries possessing random mutations in the PB2, PB1, PA, NP, M, and/or NS genes.

Virus isolate	Library Origin	HA titer (2^n)[1]	Influenza viral gene							
			**PB2**	**PB1**	**PA**	**HA (H3 numbering)**	**NP**	**NA**	**M**[2]	**NS**[3]
**UW-PR8**	-	7	n/a	n/a	n/a	n/a	n/a	n/a	n/a	n/a
**#50**	Mixed[4]	8.5	M202L/ F323L			M476I	R293M			
**#94**	Mixed	8.5	M202L/ F323L			F252I	I116L	L55S		
**#134**	Mixed	8.5	M202L/ F323L							A223E
**#154**	Mixed	8.5~9	M202L/ F323L			L182V				
**#191**	PB1	8.5		E112G (PB1-F2 R81G)[5]						
**#209**	PB1	8.5~9		R54I		E136D/ Q179L/ A194V				
**#219**	PB1	9		I667T/M714T		K162E				
**#312**	Mixed	8.5~9				L182V	I116L			R140Q
**#320**	Mixed	8.5				L182V				
**#329**	Mixed	9	M202L/ F323L			L182V				
**#344**	Mixed	8.5~9	M66R			L182V				
**#347**	Mixed	9	M202L/ F323L			L182V				
**#387**	Mixed	9	M202L/ F323L	M507V/ V644A						
**#398**	Mixed	8.5	I504V			L182V	R74K/ N417D			A30P
**#540**	PB1	8.5		E112G (PB1-F2-R81G)		K162E				S161T
**#543**	PB1	8.5		I667T						
**#545**	PB1	8.5		M40L/G180W		K449E				
**#548**	PB1	8.5~9		E112G (PB1-F2-R81G)/ L624V		K162E				S161T
**#571**	PB1	9~9.5		E112G (PB1-F2-R81G)						
**#572**	PB1	8.5		E112G (PB1-F2 R81G)						
**#573**	PB1	8.5		E112G (PB1-F2-R81G)						
**#582**	PB1	8.5~9		M40L/G180W						S161T

**#852**	Mixed	9~9.5	M202L/ F323L/ M243I	R54I						
**#965**	Mixed	8.5~9	M202L/ F323L		F105C	V184I			P90S	
**#981**	Mixed	8.5~9	M202L/ F323L	Q247H						
**#993**	Mixed	8.5~9	M202L/ F323L				N224I			
**#999**	Mixed	8.5~9	I504V			M476I	R74K/ N417D			
**#1005**	Mixed	9~9.5	M202L/ F323L	V644A	R401K	M476I				T49A
**#1007**	Mixed	8.5	I504V	V644A		F252I	M371V			
**#1014**	Mixed	8.5	I504V	T59I/G62A/ A63P/V644A/ N694K/L695T		M476I	R74K/ N417D	A265V		
**#1016**	Mixed	8.5~9	I504V			M476I				
**#1042**	Mixed	8.5~9	I504V	E75V/D76G/ E78P/P79V/S 80G/V644A/E 697P/F699L/ F700L/ P701H/ S702R/Y705T		F252I	R74K			
**#1043**	Mixed	8.5~9	I504V			L182V	R74K			
**#1045**	Mixed	9	M202L/ F323L	V644A		F252I				
**#1404**	PB1	8.5	I57V/ T58G/ A59V/ K61Q/ E677D/ D678E/ P679M	E112G (PB1- F2-R81G)/ S713C						
**#1408**	PB1	8.5		M40L/G180W						S161T

[1]HA titers were measured by HA assay with turkey red blood cells; [2]All amino acid changes localize to the M1 protein; [3]All amino acid changes localize to the NS1 protein; [4]The nine individual virus libraries were passaged twice in MDCK cells; then, aliquots of the nine libraries were combined, and the resulting ‘mixed library’ was passaged 10 more times in MDCK cells (see Figure S1); [5]The respective nucleotide change causes an E112G mutation in PB1 and an R81G mutation in the overlapping PB1-F2 protein.

Supplementary Table 2. Potentially yield-enhancing mutations identified through literature searches.

Test virus	Amino acid mutation	Phenotypic consequences	Reference	PR8 amino acid
**A/California/04/2009 (H1N1)**	PB2-E158G, NP-D101G	Altered polymerase activity and virus yield in MDCK cells	1	PB2-E158, NP-N101
**A/Tennessee/ 1-560/2009 (H1N1)**	PB2-E158A, PA-L295P, NP-H289Y	Altered polymerase activity and virus yield in MDCK cells	1	PB2-E158, PA-P295, NP-Y289
**A/seal/Massachusetts/ 1/1980 (H7N7)**	PB2-D701N/S714R, NP-N319K	Altered polymerase activity and virus yield in mammalian cells (Vero, LA-4, A549)	2	PB2-D701/N714, NP-N319
**A/Hong Kong/1/68 (H3N2)**	PB2-D701N	Altered polymerase activity and virus yield in mammalian cells (Vero, M1, A549)	3	PB2-D701
**A/duck/Guangxi/35/ 2001 (H5N1)**	PB2-D701N	Altered virus replication in mice	4	PB2-D701
**A/PR/8/34 (H1N1)**	PB2-I504V, PA- I550L	Altered polymerase activity and virus replication in mice	5	PB2-I504, PA-I550
**A/quail/Hong Kong/G1/1997 (H9N2)**	PB2-D253N/Q591K	Altered polymerase activity and replication in MDCK, normal human bronchioepithelial cell (NHBE) and mice	6	PB2-D253/Q591
**A/chicken/Yamaguchi/ 7/2004 (H5N1)**	PB2-D256G	Altered polymerase activity in 293T cells	7	PB2-D256
**A/Indonesia/UT3006/ 2005 (H5N1)**	PB2-591K	Altered polymerase activity and virus replication in NHBE cells	8	PB2-Q591
**A/Vietnam/1203/2004 (H5N1)**	PB2-368R/391E/447H/627E	Altered polymerase activity and virus replication in mice and ferrets	9	PB2-R368/E391/Q447/K627
**A/chicken/Vietnam/C58/ 2004 (H5N1)**	PB2-368R/391E/447H/627E	Altered polymerase activity and virus replication in mice and ferrets	9	PB2-R368/E391/Q447/K627
**A/Cambodia/P0322095/ 2005 (H5N1)**	PB1-L473V/L598P	Altered polymerase activity and replication in MDCK and A549 cells	10	PB1-L473/L598
**A/Hong Kong/156/1997 (H5N1)**	PB1-F2-N66S	Altered virus replication in MDCK cells and virulence in mice	11	PB1-F2-N66
**A/Vietnam/1203/2004 (H5N1)**	PB1-F2-N66S	Altered virus replication in murine cells and virulence in mice	12	PB1-F2-N66
**A/duck/Fujian/01/2002 (H5N1)**	PA-149P/266R/357K/515T	Altered polymerase activity in 293T cells	13	PA-149S/266R/357T/515T
**A/aquatic bird/Korea/W81/2005 (H5N2)**	PA-T97I	Altered polymerase activity and replication efficiency in mice and cell culture	14	PA-T97
**Genomic signatures of human versus avian influenza A viruses**	PB2-A44S	Computational predictions (PB2-R368K, PA-K142N and PA-S421I were also shown to increase virulence in mice)	15,16	PB2-A44
	PB2-R368K			PB2-R368
	PB2-V613T			PB2-A613
	PB2-A661T			PB2-A661

	PB1-R327K			PB2-R327
	PB1-V336I			PB1-V336
	PB1-F2-K73R			PB1-F2-K73
	PB1-F2-V76A			PB1-F2-V76
	PB1-F2-R79Q			PB1-F2-R79
	PB1-F2-L82S			PB1-F2-L82
	PB1-F2-E87G			PB1-F2-E87
	PA-K142N			PA-K142
	PA-S225C			PA-S225
	PA-K356R			PA-K356
	PA-S421I			PA-S421
	NP-R293K			NP-R293
	NP-E372D			NP-E372
	NP-R422K			NP-R422
	NP-T442A			NP-T442
	NP-D455E			NP-D455
**A/PR/8/34 (H1N1)**	M-V97A/Y100H	Altered virus replication in MDCK cells	17	M-V97/Y100
**A/PR/8/34 (H1N1)**	NS1-K55E	Altered virus replication in MDCK cells	18	NS1-K55

Supplementary Table 3. Statistical analysis of viral titers of potentially yield-enhancing mutations identified in the literature.

Statistical significance of data shown in Supplementary Figure 2a (viral titer)

Comparison	Hours post-infection[1]		
	12	24	48
UW-PR8 / PB2-E158G			
UW-PR8 / PB2-E158A			
UW-PR8 / PB2-E158G+NP-N101G			
UW-PR8 / PB2-D253N/Q591K			
UW-PR8 / PB2-D256G			
UW-PR8 / PB2-E391Q			*P*<0.005
UW-PR8 / PB2-I504V+PA-I550L			*P*<0.005
UW-PR8 / PB2-Q591K			
UW-PR8 / PB2-A613T			
UW-PR8 / PB2-A661T			
UW-PR8 / PB2-D701N/S714R+NP-N319K			
UW-PR8 / PB2-D701N			
UW-PR8 / PB2-A44S			

Statistical significance of data shown in Supplementary Figure 2b (viral titer)

Comparison	Hours post-infection[1]		
	12	24	48
UW-PR8 / PB1-R327K			
UW-PR8 / PB1-V336I			
UW-PR8 / PB1-L473V/L598P			

UW-PR8 / PB1-F2 N66S			*P*<0.005
UW-PR8 / PB1-F2 K73R			*P*<0.005
UW-PR8 / PB1-F2 V76A			
UW-PR8 / PB1-F2 R79Q			
UW-PR8 / PB1-F2 L82S			
UW-PR8 / PB1-F2 E87Q			

Statistical significance of data shown in Supplementary Figure 2c (viral titer)

Comparison	Hours post-infection[1]		
	12	24	48
UW-PR8 / PA-T97I			
UW-PR8 / PA-K142N			
UW-PR8 / PA-S225C			*P*<0.005
UW-PR8 / PA-S149P/T357K			*P*<0.005
UW-PR8 / PA-K356R			*P*<0.005
UW-PR8 / PA-S421I			
UW-PR8 / PB2-I504V+PA-I550L			*P*<0.005

Statistical significance of data shown in Supplementary Figure 2d (viral titer)

Comparison	Hours post-infection[1]		
	12	24	48
UW-PR8 / NP-R293K			*P*<0.005
UW-PR8 / NP-E372D			
UW-PR8 / NP-R422K			
UW-PR8 / NP-T442A			*P*<0.05
UW-PR8 /			

NP-D455E			
UW-PR8 / NP-N101G + PB2-E158G			
UW-PR8 / NP-N319K + PB2-D701N/S714R			
UW-PR8 / M1-V97A/Y100H			
UW-PR8 / NS1-K55E			

[1]*p*-values were listed if the titer of the second virus listed in the comparison was significantly higher than that of the first virus listed.

Note: Blank fields indicate that the titer of the second virus listed in the comparison was not significantly higher than that of the first virus listed.

Supplementary Table 4. List of mutations selected for further studies of UW-PR8_Indo09 virus yield.

Viral protein	Amino acid mutations potentially conferring high-yield properties to UW-PR8_Indo09 virus
**PB2**	M66R, M202L/F323L, E391Q, I504V, Q591K, A613T
**PB1**	M40L/G180W, R54I, E112G[1], Q247H, M507V/V644A, I667T/M714T
**PB1-F2**	R81G[1], N66S, K73R
**PA**	K142N, S149P/T357K, S225C, K356R, R401K, I550L
**NP**	R74K, R74K/N417D, I116L, R293M, R293K, R422K, T442A
**M1**	V97A/Y100H
**NS1**	A30P, T49A, K55E, R140Q, S161T, A223E

[1]These amino acid changes are caused by the same nucleotide mutation.

Supplementary Table 5. Mutations in high-yield viruses isolated after sequential passages of virus mixtures in Vero cells.

Virus Isolate	HA titer (2^n)	Mutations in viral proteins					
		**PB2**	**PB1**	**PA**	**NP**	**M1**	**NS1**
**UW-PR8**	6.5	n/a	n/a	n/a	n/a	n/a	n/a
**#2**	9	I504V	M40L/ G180W	K142N	I116L	WT	A30P
**#8**	9~9.5	I504V	M40L/ G180W	R401K	I116L	WT	A30P/ R118K
**#11**	9	E391Q	M40L/ G180W	K142N	R422K	WT	A30P/ R118K
**#16**	9~9.5	E391Q	M40L/ G180W	I30T/E31K/ K142N	R74K/ S377N	WT	S161T
**#74**	9	I504V	M40L/ G180W	S225C	I116L	WT	W187G(NS2-N29K)[1]/ A223E
**#116**	9~9.5	I504V	M40L/ G180W/ N641D	K142N	I116L	V97A/ Y100H	V136M/ S161T
**#144**	9	I504V	M40L/ G180W	K356R	R422K	V97A/ Y100H	K55E
**#187**	9	M202L/ F323L	M40L/ G180W	K142N	I116L	V97A/ Y100H	K55E
**#190**	9	M202L/ F323L	M40L/ G180W	K356R	I116L/ P318S	V97A/ Y100H	K55E
**#194**	9	M202L/ F323L	Q247H	K356R	R74K	V97A/ Y100H	K55E
**#202**	9	M202L/ F323L	M507V/ V644A	K356R	R422K	V97A/ Y100H	K55E
**#205**	9	M202L/ F323L	M507V/ V644A	K356R	R422K	V97A/ Y100H	K55E
**#208**	9~9.5	M202L/ F323L	M40L/ G180W	K356R	R422K	V97A/ Y100H	K55E
**#212**	9	M202L/ F323L	Q247H	K356R	I116L	V97A/ Y100H	K55E
**#213**	9~9.5	M202L/ F323L	M40L/ G180W	K356R	I116L	V97A/ Y100H	K55E
**#214**	9~9.5	M202L/ F323L	M40L/ G180W	K356R/ H535L	R74K	V97A/ Y100H	K55E

[1]The respective mutation caused a W187G mutation in NS1 and an N29K mutation in the overlapping NS2 protein.

Supplementary Table 6. Amino acid changes of high-yield candidates generated by using reverse genetics.

#	Virus stock titer (PFU/ml)	PB2	PB1	PA	NP	M1	NS1
**UW-PR8**	$1.8x10^{7}$	WT	WT	WT	WT	WT	WT
**HY#1_Indo09**	$3.6x10^{8}$	I504V	M40L/ G180W	R401K	I116L	WT	A30P/ R118K
**HY#2_Indo09**	$1.6x10^{8}$	E391Q	M40L/ G180W	I30T/E31K/ K142N	R74K/ S377N	WT	S161T
**HY#3_Indo09**	$1.1x10^{7}$	I504V	M40L/ G180W	K142N	I116L	V97A/ Y100H	V136M/ S161T
**HY#4_Indo09**	$8x10^{6}$	M202L/ F323L	M40L/ G180W	K356R	I116L	V97A/ Y100H	K55E
**HY#5_Indo09**	$1.8x10^{8}$	M202L/ F323L	M40L/ G180W	K356R	R422K	WT	K55E
**HY#6_Indo09**	$6.1x10^{8}$	I504V	M40L/ G180W	K142N	I116L	WT	V136M/ S161T
**HY#7_Indo09**	$1.9x10^{8}$	M202L/ F323L	M40L/ G180W	K356R	I116L	WT	K55E

Supplementary Table 7. Statistical analysis of viral and HA titers of HY#1-7 high-yield candidates in Vero cells**.**

Statistical significance of data shown in Figure 2a (viral titer)

Comparison	Hours post-infection[1]			
	24	48	72	96
UW-PR8_Indo09/ HY#1_Indo09	*P*<0.005	*P*<0.005	*P*<0.005	*P*<0.005
UW-PR8_Indo09/ HY#2_Indo09	*P*<0.005	*P*<0.005	*P*<0.005	*P*<0.005
UW-PR8_Indo09/ HY#3_Indo09				*P*<0.05
UW-PR8_Indo09/ HY#4_Indo09				
UW-PR8_Indo09/ HY#5_Indo09			*P*<0.005	*P*<0.005
UW-PR8_Indo09/ HY#6_Indo09	*P*<0.005		*P*<0.005	*P*<0.005
UW-PR8_Indo09/ HY#7_Indo09				*P*<0.005

Statistical significance of data shown in Figure 2a (HA titer)

Comparison	Hours post-infection[1]			
	24	48	72	96
UW-PR8_Indo09/ HY#1_Indo09		*P*<0.005	*P*<0.005	*P*<0.005
UW-PR8_Indo09/ HY#2_Indo09	*P*<0.005	*P*<0.005	*P*<0.005	*P*<0.005
UW-PR8_Indo09/ HY#3_Indo09				*P*<0.05
UW-PR8_Indo09/ HY#4_Indo09				
UW-PR8_Indo09/ HY#5_Indo09			*P*<0.005	*P*<0.005
UW-PR8_Indo09/ HY#6_Indo09		*P*<0.005	*P*<0.005	*P*<0.005
UW-PR8_Indo09/ HY#7_Indo09				

Statistical significance of data shown in Figure 2b (viral titer)

Comparison	Hours post-infection[1]					
	12	24	36	48	60	72
UW-PR8_Indo09 / HY#1_Indo09	*P*<0.005	*P*<0.005	*P*<0.005	*P*<0.005	*P*<0.005	*P*<0.005
UW-PR8_Indo09 / HY#1+C4U_Indo09	*P*<0.005	*P*<0.005	*P*<0.005	*P*<0.005	*P*<0.005	*P*<0.005
HY#1_Indo09 HY#1+C4U_Indo09	*P*<0.005		*P*<0.005			

Statistical significance of data shown in Figure 2b (HA titer)

Comparison	Hours post-infection[1]					
	12	24	36	48	60	72
UW-PR8_Indo09 / HY#1_Indo09		*P*<0.005	*P*<0.005	*P*<0.005	*P*<0.005	*P*<0.005
UW-PR8_Indo09 / HY#1+C4U_Indo09		*P*<0.005	*P*<0.005	*P*<0.005	*P*<0.005	*P*<0.005
HY#1_Indo09 HY#1+C4U_Indo09		*P*<0.05	*P*<0.005	*P*<0.01		

[1] *p*-values were listed if the titer of the second virus listed in the comparison was significantly higher than that of the first virus listed.

Note: Blank fields indicate that the titer of the second virus listed in the comparison was not significantly higher than that of the first virus listed.

Supplementary Table 8. Junction sites of chimeric HA and NA proteins.

Junction sites of chimeric HA proteins

Strain	N-terminal junction site*	C-terminal junction site
**A/chicken/Indonesia/NC/2009 (H5N1)**	***...CALAAADA***/DQICIGYHANNSTEQ…	...REEISGVKLESIGTYQ***/ILAIYSTVAS...***
**A/Vietnam/1203/2004 (H5N1)**	***...CALAAADA***/DQICIGYHANNSTEQ…	...REEISGVKLESIGIYQ***/ILAIYSTVAS…***
**A/Hubei/1/2010 (H5N1)**	***...CALAAADA***/DHICIGYHANNSTEQ…	...REEISGVKLESIGIYQ***/ILAIYSTVAS...***
**A/Egypt/N03072/2010 (H5N1)**	***...CALAAADA***/DQICIGYHANNSTEQ…	...REEISGVKLESIGTYQ***/ILAIYSTVAS...***
**A/Indonesia/05/2005 (H5N1)**	***...CALAAADA***/DQICIGYHANNSTEQ…	...REEISGVKLESIGTYQ***/ILAIYSTVAS...***
**A/Anhui/1/2013 (H5N1)**	***...CALAAADA***/DKICLGHHAVSNGTK…	...IQIDPVKLSSGYKDV***/ILAIYSTVAS...***
**X-181 (Derived from A/California/07/2009, H1N1)**	***...CALAAADA***/DTLCIGYHANNSTDT…	...REEIDGVKLESTRIYQ***/ILAIYSTVAS...***
**X-223A (Derived from A/Texas/50/2012, H3N2)**	***...CALAAADA***/QKLPGNDNSTATLCL…	...FQIKGVELKSGYKDW***/ILAIYSTVAS...***

Junction sites of chimeric NA proteins

Strain	N-terminal junction site	C-terminal junction site
**A/chicken/Indonesia/NC/2009 (H5N1)**	***...LQIGNIISIWIS/***HSIQKGNQHQAES…	...SWPDGAELPFTIDK***/TAG(stop codon)…***
**A/Vietnam/1203/2004 (H5N1)**	***...LQIGNIISIWIS/***HSIHTGNQHQSEP…	...SWPDGAELPFTIDK***/TAG(stop codon)…***
**A/Hubei/1/2010 (H5N1)**	***...LQIGNIISIWIS/***HSIQTGNQHQTEP…	...SWPDGAELPFTIDK***/TAG(stop codon)…***
**A/Egypt/N03072/2010 (H5N1)**	***...LQIGNIISIWIS/***HSIQTGNQCQDEP…	...SWPDGAELPFTIDK***/TAG(stop codon)…***
**A/Indonesia/05/2005 (H5N1)**	***...LQIGNIISIWIS/***HSIQTGNQHQAES…	...SWPDGAELPFTIDK***/TAG(stop codon)…***
**A/Anhui/1/2013 (H5N1)**	***...LQIGNIISIWIS/***HLKPGCNCSHSQP…	...WNWPDGAKIEYFL***/TAG(stop codon)…***
**X-181 (Derived from A/California/07/2009, H1N1)**	***...LQIGNIISIWIS/***HSIQLGNQNQIET…	...SWPDGAELPFTIDK***/TAG(stop codon)…***
**X-223A (Derived from A/Texas/50/2012, H3N2)**	***...LQIGNIISIWIS/***HFKQYEFNSPPNN…	...SWPDGADLNLMPI***/TAG(stop codon)…***

*PR8 virus sequences are shown in bold face and italicized.

Supplementary Table 9. Statistical analysis of viral and HA titers of HA/NA chimeric viruses in Vero cells.

Statistical significance of data shown in Figure 3a (viral titer)

Comparison	Hours post-infection[1]					
	12	24	36	48	60	72
UW-PR8_Indo09 / UW-PR8_$Indo09_{Chim}$	*P*<0.005	*P*<0.005	*P*<0.005	*P*<0.005	*P*<0.005	*P*<0.005

Statistical significance of data shown in Figure 3a (HA titer)

Comparison	Hours post-infection[1]					
	12	24	36	48	60	72
UW-PR8_Indo09 / UW-PR8_$Indo09_{Chim}$		*P*<0.005	*P*<0.005	*P*<0.005	*P*<0.005	*P*<0.005

Statistical significance of data shown in Figure 3b (viral titer)

Comparison	Hours post-infection[1]					
	12	24	36	48	60	72
UW-PR8_Indo09 / PR8-HY_Indo09	*P*<0.005	*P*<0.005	*P*<0.005	*P*<0.005	*P*<0.005	*P*<0.005
UW-PR8_Indo09 / PR8-HY_$Indo09_{Chim}$	*P*<0.005	*P*<0.005	*P*<0.005	*P*<0.005	*P*<0.005	*P*<0.005
PR8-HY_Indo09 / PR8-HY_$Indo09_{Chim}$						*P*<0.01

Statistical significance of data shown in Figure 3b (HA titer)

Comparison	Hours post-infection[1]					
	12	24	36	48	60	72
UW-PR8_Indo09 / PR8-HY_Indo09		*P*<0.005	*P*<0.005	*P*<0.005	*P*<0.005	*P*<0.005
UW-PR8_Indo09 / PR8-HY_$Indo09_{Chim}$		*P*<0.005	*P*<0.005	*P*<0.005	*P*<0.005	*P*<0.005
PR8-HY_Indo09 / PR8-HY_$Indo09_{Chim}$			*P*<0.005	*P*<0.005	*P*<0.005	*P*<0.005

[1]*p*-values were listed if the titer of the second virus listed in the comparison was significantly higher than that of the first virus listed.

Note: Blank fields indicate that the titer of the second virus listed in the comparison was not significantly higher than that of the first virus listed.

Supplementary Table 10. Statistical analysis of viral and HA titers of PR8-HY vaccine candidate viruses propagated in Vero cells.

Statistical significance of data shown in Figure 4a (viral titer)

Comparison	Hours post-infection[1]			
	24	36	48	72
UW-PR8_VN04 / PR8-HY_VN04	*P*<0.005	*P*<0.005	*P*<0.005	*P*<0.005
UW-PR8_VN04 / PR8-HY_VN04$_{Chim}$	*P*<0.005	*P*<0.005	*P*<0.005	*P*<0.005
PR8-HY_VN04 / PR8-HY_VN04$_{Chim}$				

Statistical significance of data shown in Figure 4a (HA titer)

Comparison	Hours post-infection[1]			
	24	36	48	72
UW-PR8_VN04 / PR8-HY_VN04	*P*<0.005	*P*<0.005	*P*<0.005	*P*<0.005
UW-PR8_VN04 / PR8-HY_VN04$_{Chim}$	*P*<0.005	*P*<0.005	*P*<0.005	*P*<0.005
PR8-HY_VN04 / PR8-HY_VN04$_{Chim}$				

Statistical significance of data shown in Figure 4b (viral titer)

Comparison	Hours post-infection[1]			
	24	36	48	72
UW-PR8_Hubei10 / PR8-HY_Hubei10	*P*<0.005	*P*<0.005	*P*<0.005	*P*<0.005
UW-PR8_Hubei10 / PR8-HY_Hubei10$_{Chim}$	*P*<0.005	*P*<0.005	*P*<0.005	*P*<0.005
PR8-HY_Hubei10 / PR8-HY_Hubei10$_{Chim}$	*P*<0.005	*P*<0.005	*P*<0.05	

Statistical significance of data shown in Figure 4b (HA titer)

Comparison	Hours post-infection[1]			
	24	36	48	72
UW-PR8_Hubei10 / PR8-HY_Hubei10		*P*<0.005	*P*<0.005	*P*<0.005
UW-PR8_Hubei10 / PR8-HY_Hubei10$_{Chim}$		*P*<0.005	*P*<0.005	*P*<0.005
PR8-HY_Hubei10 / PR8-HY_Hubei10$_{Chim}$		*P*<0.005	*P*<0.005	

Statistical significance of data shown in Figure 4c (viral titer)

Comparison	Hours post-infection[1]				
	24	36	48	72	96
UW-PR8_Egypt10 / PR8-HY_Egypt10	P<0.005	P<0.005	P<0.005	P<0.005	P<0.005
UW-PR8_Egypt10 / PR8-HY_Egypt10$_{Chim}$	P<0.005	P<0.005	P<0.005	P<0.005	P<0.005
PR8-HY_Egypt10 / PR8-HY_Egypt10$_{Chim}$		P<0.005			

Statistical significance of data shown in Figure 4c (HA titer)

Comparison	Hours post-infection[1]				
	24	36	48	72	96
UW-PR8_Egypt10 / PR8-HY_Egypt10		P<0.005	P<0.005	P<0.005	P<0.005
UW-PR8_Egypt10 / PR8-HY_Egypt10$_{Chim}$		P<0.005	P<0.005	P<0.005	P<0.005
PR8-HY_Egypt10 / PR8-HY_Egypt10$_{Chim}$		P<0.05	P<0.01		

Statistical significance of data shown in Figure 4d (viral titer)

Comparison	Hours post-infection[1]			
	24	36	48	72
UW-PR8_Indo05 / PR8-HY_Indo05	P<0.005	P<0.005	P<0.005	P<0.005
UW-PR8_Indo05 / PR8-HY_Indo05$_{Chim}$	P<0.005	P<0.005	P<0.005	P<0.005
PR8-HY_Indo05 / PR8-HY_Indo05$_{Chim}$	P<0.005	P<0.005		

Statistical significance of data shown in Figure 4d (HA titer)

Comparison	Hours post-infection[1]			
	24	36	48	72
UW-PR8_Indo05 / PR8-HY_Indo05	P<0.005	P<0.005	P<0.005	P<0.005
UW-PR8_Indo05 / PR8-HY_Indo05$_{Chim}$	P<0.005	P<0.005	P<0.005	P<0.05
PR8-HY_Indo05 / PR8-HY_Indo05$_{Chim}$	P<0.005	P<0.005		

Statistical significance of data shown in Figure 4e (viral titer)

Comparison	Hours post-infection[1]				
	12	24	48	72	96
UW-PR8_Anhui13 / PR8-HY_Anhui13	*P*<0.005	*P*<0.005	*P*<0.005	*P*<0.005	*P*<0.005
UW-PR8_Anhui13 / PR8-HY_Anhui13$_{Chim}$	*P*<0.005	*P*<0.005	*P*<0.005	*P*<0.005	*P*<0.005
PR8-HY_Anhui13 / PR8-HY_Anhui13$_{Chim}$	*P*<0.005	*P*<0.005	*P*<0.005	*P*<0.01	*P*<0.005

Statistical significance of data shown in Figure 4e (HA titer)

Comparison	Hours post-infection[1]				
	12	24	48	72	96
UW-PR8_Anhui13 / PR8-HY_Anhui13			*P*<0.005	*P*<0.005	*P*<0.005
UW-PR8_Anhui13 / PR8-HY_Anhui13$_{Chim}$			*P*<0.005	*P*<0.005	*P*<0.005
PR8-HY_Anhui13 / PR8-HY_Anhui13$_{Chim}$			*P*<0.005		*P*<0.005

Statistical significance of data shown in Figure 4f (viral titer)

Comparison	Hours post-infection[1]						
	24	48	72	96	120	144	168
X-181 / PR8-HY_X-181			*P*<0.005	*P*<0.005	*P*<0.005	*P*<0.005	*P*<0.005
X-181 / PR8-HY_X-181$_{Chim}$				*P*<0.005	*P*<0.005	*P*<0.005	*P*<0.005
PR8-HY_X-181 / PR8-HY_X-181$_{Chim}$							

Statistical significance of data shown in Figure 4f (HA titer)

Comparison	Hours post-infection[1]						
	24	48	72	96	120	144	168
X-181 / PR8-HY_X-181				*P*<0.005	*P*<0.005	*P*<0.005	*P*<0.005
X-181 / PR8-HY_X-181$_{Chim}$						*P*<0.005	*P*<0.005
PR8-HY_X-181 / PR8-HY_X-181$_{Chim}$							

Statistical significance of data shown in Figure 4g (viral titer)

Comparison	Hours post-infection[1]						
	24	36	48	60	72	84	96
X-223A / PR8-HY_X-223A	*P*<0.005	*P*<0.005	*P*<0.005	*P*<0.005	*P*<0.005	*P*<0.005	*P*<0.005

Statistical significance of data shown in Figure 4g (HA titer)

Comparison	Hours post-infection[1]						
	24	36	48	60	72	84	96
X-223A / PR8-HY_X-223A				*P*<0.005	*P*<0.005	*P*<0.005	*P*<0.005

[1]*p*-values were listed if the titer of the second virus listed in the comparison was significantly higher than that of the first virus listed.

Note: Blank fields indicate that the titer of the second virus listed in the comparison was not significantly higher than that of the first virus listed.

Supplementary Table 11. Statistical analysis of viral and HA titers of PR8-HY vaccine candidate viruses propagated in MDCK cells.

Statistical significance of data shown in Figure 5a (viral titer)

Comparison	Hours post-infection[1]			
	12	24	36	48
UW-PR8_Indo09 / PR8-HY_Indo09	*P*<0.005	*P*<0.005	*P*<0.005	*P*<0.005
UW-PR8_Indo09 / PR8-HY_Indo09$_{Chim}$	*P*<0.005	*P*<0.005	*P*<0.005	*P*<0.005
PR8-HY_Indo09 / PR8-HY_Indo09$_{Chim}$				

Statistical significance of data shown in Figure 5a (HA titer)

Comparison	Hours post-infection[1]			
	12	24	36	48
UW-PR8_Indo09 / PR8-HY_Indo09		*P*<0.005	*P*<0.005	*P*<0.005
UW-PR8_Indo09 / PR8-HY_Indo09$_{Chim}$		*P*<0.005	*P*<0.005	*P*<0.005
PR8-HY_Indo09 / PR8-HY_Indo09$_{Chim}$			*P*<0.01	

Statistical significance of data shown in Figure 5b (viral titer)

Comparison	Hours post-infection[1]			
	12	24	36	48
UW-PR8_VN04 / PR8-HY_VN04	*P*<0.005	*P*<0.01		
UW-PR8_VN04 / PR8-HY_VN04$_{Chim}$	*P*<0.005	*P*<0.05		
PR8-HY_VN04 / PR8-HY_VN04$_{Chim}$				

Statistical significance of data shown in Figure 5b (HA titer)

Comparison	Hours post-infection[1]			
	12	24	36	48
UW-PR8_VN04 / PR8-HY_VN04		*P*<0.005	*P*<0.005	*P*<0.005
UW-PR8_VN04 / PR8-HY_VN04$_{Chim}$		*P*<0.005	*P*<0.005	*P*<0.005
PR8-HY_VN04 / PR8-HY_VN04$_{Chim}$				

Statistical significance of data shown in Figure 5c (viral titer)

Comparison	Hours post-infection[1]			
	12	24	36	48
UW-PR8_Hubei10 / PR8-HY_Hubei10	*P*<0.005	*P*<0.005	*P*<0.05	*P*<0.05
UW-PR8_Hubei10 / PR8-HY_Hubei10$_{Chim}$	*P*<0.005	*P*<0.005	*P*<0.01	*P*<0.05
PR8-HY_Hubei10 / PR8-HY_Hubei10$_{Chim}$	*P*<0.005	*P*<0.005		

Statistical significance of data shown in Figure 5c (HA titer)

Comparison	Hours post-infection[1]			
	12	24	36	48
UW-PR8_Hubei10 / PR8-HY_Hubei10				
UW-PR8_Hubei10 / PR8-HY_Hubei10$_{Chim}$		*P*<0.005	*P*<0.005	
PR8-HY_Hubei10 / PR8-HY_Hubei10$_{Chim}$		*P*<0.005		

Statistical significance of data shown in Figure 5d (viral titer)

Comparison	Hours post-infection[1]			
	12	24	36	48
UW-PR8_Egypt10 / PR8-HY_Egypt10	*P*<0.005	*P*<0.005	*P*<0.005	
UW-PR8_Egypt10 / PR8-HY_Egypt10$_{Chim}$	*P*<0.005	*P*<0.005	*P*<0.005	
PR8-HY_Egypt10 / PR8-HY_Egypt10$_{Chim}$				

Statistical significance of data shown in Figure 5d (HA titer)

Comparison	Hours post-infection[1]			
	12	24	36	48
UW-PR8_Egypt10 / PR8-HY_Egypt10		*P*<0.005	*P*<0.005	*P*<0.005
UW-PR8_Egypt10 / PR8-HY_Egypt10$_{Chim}$		*P*<0.005	*P*<0.005	*P*<0.005
PR8-HY_Egypt10 / PR8-HY_Egypt10$_{Chim}$				

Statistical significance of data shown in Figure 5e (viral titer)

Comparison	Hours post-infection[1]			
	12	24	36	48
UW-PR8_Indo05 / PR8-HY_Indo05	*P*<0.005	*P*<0.005	*P*<0.005	*P*<0.005
UW-PR8_Indo05 / PR8-HY_Indo05$_{Chim}$	*P*<0.005	*P*<0.005	*P*<0.005	*P*<0.005
PR8-HY_Indo05 / PR8-HY_Indo05$_{Chim}$				

Statistical significance of data shown in Figure 5e (HA titer)

Comparison	Hours post-infection[1]			
	12	24	36	48
UW-PR8_Indo05 / PR8-HY_Indo05		*P*<0.005	*P*<0.005	*P*<0.005
UW-PR8_Indo05 / PR8-HY_Indo05$_{Chim}$		*P*<0.005	*P*<0.005	*P*<0.005
PR8-HY_Indo05 / PR8-HY_Indo05$_{Chim}$		*P*<0.05	*P*<0.05	*P*<0.01

Statistical significance of data shown in Figure 5f (viral titer)

Comparison	Hours post-infection[1]			
	12	24	36	48
UW-PR8_Anhui13 / PR8-HY_Anhui13	*P*<0.005	*P*<0.005	*P*<0.005	*P*<0.005
UW-PR8_Anhui13 / PR8-HY_Anhui13$_{Chim}$	*P*<0.005	*P*<0.005	*P*<0.005	*P*<0.005
PR8-HY_Anhui13 / PR8-HY_Anhui13$_{Chim}$	*P*<0.005	*P*<0.005	*P*<0.005	*P*<0.005

Statistical significance of data shown in Figure 5f (HA titer)

Comparison	Hours post-infection[1]			
	12	24	36	48
UW-PR8_Anhui13 / PR8-HY_Anhui13		*P*<0.005	*P*<0.005	*P*<0.005
UW-PR8_Anhui13 / PR8-HY_Anhui13$_{Chim}$		*P*<0.005	*P*<0.005	*P*<0.005
PR8-HY_Anhui13 / PR8-HY_Anhui13$_{Chim}$		*P*<0.005	*P*<0.005	*P*<0.005

Statistical significance of data shown in Figure 5g (viral titer)

Comparison	Hours post-infection[1]			
	12	24	36	48
X-181 / PR8-HY_X-181	*P*<0.05	*P*<0.01	*P*<0.005	*P*<0.005
X-181 / PR8-HY_X-181$_{Chim}$	*P*<0.005	*P*<0.005	*P*<0.005	*P*<0.005
PR8-HY_X-181 / PR8-HY_X-181$_{Chim}$				

Statistical significance of data shown in Figure 5g (HA titer)

Comparison	Hours post-infection[1]			
	12	24	36	48
X-181 / PR8-HY_X-181				
X-181 / PR8-HY_X-181$_{Chim}$				
PR8-HY_X-181 / PR8-HY_X-181$_{Chim}$				

Statistical significance of data shown in Figure 5h (viral titer)

Comparison	Hours post-infection[1]			
	12	24	36	48
X-223A / PR8-HY_X-223A	*P*<0.005	*P*<0.005	*P*<0.005	*P*<0.005

Statistical significance of data shown in Figure 5h (HA titer)

Comparison	Hours post-infection[1]			
	12	24	36	48
X-223A / PR8-HY_X-223A			*P*<0.05	

[1] *p*-values are were if the titer of the second virus listed in the comparison was significantly higher than that of the first virus listed.

Note: Blank fields indicate that the titer of the second virus listed in the comparison was not significantly higher than that of the first virus listed.

Supplementary Table 12. Statistical analysis of viral and HA titers of PR8-HY vaccine candidate viruses propagated in embryonated chicken eggs.

Statistical significance of data shown in Figure 6a (viral titer)

Comparison	Hours post-infection[1]				
	12	24	36	48	60
UW-PR8_Indo09 / PR8-HY_Indo09		*P*<0.005	*P*<0.05		
UW-PR8_Indo09 / PR8-HY_Indo09$_{Chim}$		*P*<0.005	*P*<0.005	*P*<0.005	*P*<0.005
PR8-HY_Indo09 / PR8-HY_Indo09$_{Chim}$	*P*<0.05				

Statistical significance of data shown in Figure 6a (HA titer)

Comparison	Hours post-infection[1]				
	12	24	36	48	60
UW-PR8_Indo09 / PR8-HY_Indo09					
UW-PR8_Indo09 / PR8-HY_Indo09$_{Chim}$		*P*<0.005	*P*<0.005	*P*<0.005	*P*<0.005
PR8-HY_Indo09 / PR8-HY_Indo09$_{Chim}$		*P*<0.05			

Statistical significance of data shown in Figure 6b (viral titer)

Comparison	Hours post-infection[1]					
	12	18	24	36	48	60
UW-PR8_VN04 / PR8-HY_VN04	*P*<0.005		*P*<0.005	*P*<0.005	*P*<0.005	*P*<0.005
UW-PR8_VN04 / PR8-HY_VN04$_{Chim}$			*P*<0.005	*P*<0.005	*P*<0.005	*P*<0.005
PR8-HY_VN04 / PR8-HY_VN04$_{Chim}$	*P*<0.05	*P*<0.005				

Statistical significance of data shown in Figure 6b (HA titer)

Comparison	Hours post-infection[1]					
	12	18	24	36	48	60
UW-PR8_VN04 / PR8-HY_VN04			*P*<0.05	*P*<0.05	*P*<0.05	
UW-PR8_VN04 / PR8-HY_VN04$_{Chim}$						
PR8-HY_VN04 / PR8-HY_VN04$_{Chim}$						

Statistical significance of data shown in Figure 6c (viral titer)

Comparison	Hours post-infection[1]			
	12	24	48	60
UW-PR8_Hubei10 / PR8-HY_Hubei10		*P*<0.005	*P*<0.005	*P*<0.005
UW-PR8_Hubei10 / PR8-HY_Hubei10$_{Chim}$		*P*<0.005	*P*<0.005	*P*<0.005
PR8-HY_Hubei10 / PR8-HY_Hubei10$_{Chim}$				

Statistical significance of data shown in Figure 6c (HA titer)

Comparison	Hours post-infection[1]			
	12	24	48	60
UW-PR8_Hubei10 / PR8-HY_Hubei10			*P*<0.005	*P*<0.005
UW-PR8_Hubei10 / PR8-HY_Hubei10$_{Chim}$		*P*<0.005	*P*<0.005	*P*<0.005
PR8-HY_Hubei10 / PR8-HY_Hubei10$_{Chim}$				

Statistical significance of data shown in Figure 6d (viral titer)

Comparison	Hours post-infection[1]				
	12	24	36	48	60
UW-PR8_Egypt10 / PR8-HY_Egypt10	*P*<0.005	*P*<0.005	*P*<0.005	*P*<0.005	
UW-PR8_Egypt10 / PR8-HY_Egypt10$_{Chim}$	*P*<0.005	*P*<0.05	*P*<0.005	*P*<0.005	
PR8-HY_Egypt10 / PR8-HY_Egypt10$_{Chim}$					

Statistical significance of data shown in Figure 6d (HA titer)

Comparison	Hours post-infection[1]				
	12	24	36	48	60
UW-PR8_Egypt10 / PR8-HY_Egypt10					
UW-PR8_Egypt10 / PR8-HY_Egypt10$_{Chim}$					
PR8-HY_Egypt10 / PR8-HY_Egypt10$_{Chim}$					

Statistical significance of data shown in Figure 6e (viral titer)

Comparison	Hours post-infection[1]				
	12	24	36	48	60
UW-PR8_Indo05 / PR8-HY_Indo05		*P*<0.005	*P*<0.005	*P*<0.005	*P*<0.01
UW-PR8_Indo05 / PR8-HY_Indo05$_{Chim}$		*P*<0.005	*P*<0.005	*P*<0.005	*P*<0.005
PR8-HY_Indo05 / PR8-HY_Indo05$_{Chim}$					

Statistical significance of data shown in Figure 6e (HA titer)

Comparison	Hours post-infection[1]				
	12	24	36	48	60
UW-PR8_Indo05 / PR8-HY_Indo05		*P*<0.01		*P*<0.005	*P*<0.005
UW-PR8_Indo05 / PR8-HY_Indo05$_{Chim}$		*P*<0.01		*P*<0.005	*P*<0.005
PR8-HY_Indo05 / PR8-HY_Indo05$_{Chim}$					

Statistical significance of data shown in Figure 6f (viral titer)

Comparison	Hours post-infection[1]				
	12	24	36	48	60
UW-PR8_Anhui13 / PR8-HY_Anhui13			*P*<0.005	*P*<0.005	*P*<0.005
UW-PR8_Anhui13 / PR8-HY_Anhui13$_{Chim}$	*P*<0.005	*P*<0.005	*P*<0.005	*P*<0.005	*P*<0.005
PR8-HY_Anhui13 / PR8-HY_Anhui13$_{Chim}$	*P*<0.05	*P*<0.005	*P*<0.005		

Statistical significance of data shown in Figure 6f (HA titer)

Comparison	Hours post-infection[1]				
	12	24	36	48	60
UW-PR8_Anhui13 / PR8-HY_Anhui13			*P*<0.005	*P*<0.005	*P*<0.005
UW-PR8_Anhui13 / PR8-HY_Anhui13$_{Chim}$		*P*<0.005	*P*<0.005	*P*<0.005	*P*<0.005
PR8-HY_Anhui13 / PR8-HY_Anhui13$_{Chim}$		*P*<0.005			

Statistical significance of data shown in Figure 6g (viral titer)

Comparison	Hours post-infection[1]				
	12	24	36	48	60
X-181 / PR8-HY_X-181				*P*<0.05	
X-181 / PR8-HY_X-181$_{Chim}$			*P*<0.005	*P*<0.005	*P*<0.005
PR8-HY_X-181 / PR8-HY_X-181$_{Chim}$	*P*<0.005	*P*<0.05	*P*<0.01		*P*<0.005

Statistical significance of data shown in Figure 6g (HA titer)

Comparison	Hours post-infection[1]				
	12	24	36	48	60
X-181 / PR8-HY_X-181					
X-181 / PR8-HY_X-181$_{Chim}$			*P*<0.05		
PR8-HY_X-181 / PR8-HY_X-181$_{Chim}$		*P*<0.05	*P*<0.05		

Statistical significance of data shown in Figure 6h (viral titer)

Comparison	Hours post-infection[1]				
	12	24	36	48	60
X-223A / PR8-HY_X-223A			*P*<0.05		

Statistical significance of data shown in Figure 6h (HA titer)

Comparison	Hours post-infection[1]				
	12	24	36	48	60
X-223A / PR8-HY_X-223A					

[1] *p*-values were listed if the titer of the second virus listed in the comparison was significantly higher than that of the first virus listed.

Note: Blank fields indicate that the titer of the second virus listed in the comparison was not significantly higher than that of the first virus listed.

Supplementary Table 13. Frequency in human and avian influenza viruses of amino acid changes in PR8-HY.

Viral protein	Position and mutation	Human influenza viruses			Avian influenza viruses		
		Amino acid	Number of viruses	%	Amino acid	Number of viruses	%
PB2	I504V	I	39	0.32	I	4	0.04
		V	12213	99.65	V	9789	99.88
		S	4	0.03	Other	8	0.08
PB1	M40L	M	11689	99.78	M	10315	99.22
		L	2	0.02	L	32	0.31
		I	19	0.16	I	47	0.45
		Other	5	0.04	Other	2	0.02
	G180W	G	44	0.38	G	35	0.34
		E	11644	99.54	E	10324	99.52
		K	7	0.06	D	11	0.11
		Other	2	0.02	Other	4	0.04
PA	R401K	R	11485	99.49	R	9712	97.93
		K	56	0.49	K	197	1.99
		Other	3	0.03	Other	8	0.08
NP	I116L	I	11975	99.817	I	10645	99.34
		L	1	0.008	L	42	0.39
		V	21	0.175	V	28	0.26
					Other	1	0.01
NS1	A30P	A	11785	99.88	A	8753	99.91
		S	6	0.05	S	1	0.01
		T	3	0.03	T	3	0.03
		V	5	0.04	V	4	0.05
	R118K	R	11663	98.86	R	5960	68
		K	118	1.00	K	2804	32
		Q	14	0.12			
		Other	2	0.02	None	None	N/A

Supplementary Table 14. Database information and acknowledgments for the H7N9 and H5N1 virus HA and NA sequences used to generate the vaccine viruses in this study.

We acknowledge the authors, originating and submitting laboratories of the sequences from GISAID's EpiFlu™ Database on which this research is based.

All submitters of data may be contacted directly via the GISAID website ***www.gisaid.org***

Segment ID	Segment	Country	Collection date	Isolate name	Submitting Lab	Authors
**EPI267032**	HA	China	1-Jun-2010	A/Hubei/1/2010 (H5N1)	WHO Chinese National Influenza Center	Yu Lan,Wei Wang,Shumei Zou,Zi Li,Leying Wen,Xiaodan Li,Libo Dong,Dexin Li,Yuelong Shu
**EPI267031**	NA	China	1-Jun-2010	A/Hubei/1/2010 (H5N1)	WHO Chinese National Influenza Center	Yu Lan,Wei Wang,Shumei Zou,Zi Li,Leying Wen,Xiaodan Li,Libo Dong,Dexin Li,Yuelong Shu
**EPI255379**	HA	Egypt	7-Mar-2010	A/Egypt/N03072/2010 (H5N1)		Elassal,E.M.,Kandeel,A.,Abdelghani,A.S.,Elsayed, N.M.,Gomaa,A.A.,Younan,M.,Earhart,K.C.,Turner, M.,Pimentel,G.,Barthel,R.V.
**EPI255380**	NA	Egypt	7-Mar-2010	A/Egypt/N03072/2010 (H5N1)		Elassal,E.M.,Kandeel,A.,Abdelghani,A.S.,Elsayed, N.M.,Gomaa,A.A.,Younan,M.,Earhart,K.C.,Turner, M.,Pimentel,G.,Barthel,R.V.
**EPI376537**	HA	Indonesia	2005	A/Indonesia/05/2005 (H5N1)	Erasmus Medical Center	Herfst,S., Schrauwen,E.J., Linster,M., Chutinimitkul,S., de Wit,E., Munster,V.J., Sorrell,E.M., Bestebroer,T.M., Burke,D.F.,Smith,D.J., Rimmelzwaan,G.F., Osterhaus,A.D. and Fouchier,R.A.
**EPI376539**	NA	Indonesia	2005	A/Indonesia/05/2005 (H5N1)	Erasmus Medical Center	Herfst,S., Schrauwen,E.J., Linster,M., Chutinimitkul,S., de Wit,E., Munster,V.J., Sorrell,E.M., Bestebroer,T.M., Burke,D.F.,Smith,D.J., Rimmelzwaan,G.F., Osterhaus,A.D. and Fouchier,R.A.
**EPI439507**	HA	China	20-Mar-2013	A/Anhui/1/2013 (H7N9)	WHO Chinese National Influenza Center	
**EPI439509**	NA	China	20-Mar-2013	A/Anhui/1/2013 (H7N9)	WHO Chinese National Influenza Center	

Supplementary References

1. Ilyushina, N.A.*, et al.* Adaptation of pandemic H1N1 influenza viruses in mice. *J Virol* **84**, 8607-8616 (2010).
2. Gabriel, G.*, et al.* The viral polymerase mediates adaptation of an avian influenza virus to a mammalian host. *Proc Natl Acad Sci U S A* **102**, 18590-18595 (2005).
3. Ping, J.*, et al.* PB2 and hemagglutinin mutations are major determinants of host range and virulence in mouse-adapted influenza A virus. *J Virol* **84**, 10606-10618 (2010).
4. Li, Z.*, et al.* Molecular basis of replication of duck H5N1 influenza viruses in a mammalian mouse model. *J Virol* **79**, 12058-12064 (2005).
5. Rolling, T.*, et al.* Adaptive mutations resulting in enhanced polymerase activity contribute to high virulence of influenza A virus in mice. *J Virol* **83**, 6673-6680 (2009).
6. Mok, C.K.*, et al.* Amino acid residues 253 and 591 of the PB2 protein of avian influenza virus A H9N2 contribute to mammalian pathogenesis. *J Virol* **85**, 9641-9645 (2011).
7. Manzoor, R.*, et al.* PB2 protein of a highly pathogenic avian influenza virus strain A/chicken/Yamaguchi/7/2004 (H5N1) determines its replication potential in pigs. *J Virol* **83**, 1572-1578 (2009).
8. Yamada, S.*, et al.* Biological and structural characterization of a host-adapting amino acid in influenza virus. *PLoS Pathog* **6**, e1001034 (2010).
9. Salomon, R.*, et al.* The polymerase complex genes contribute to the high virulence of the human H5N1 influenza virus isolate A/Vietnam/1203/04. *J Exp Med* **203**, 689-697 (2006).
10. Xu, C.*, et al.* Amino acids 473V and 598P of PB1 from an avian-origin influenza A virus contribute to polymerase activity, especially in mammalian cells. *Journal of General Virology* **93**, 531-540 (2012).

11. Conenello, G.M., Zamarin, D., Perrone, L.A., Tumpey, T. & Palese, P. A single mutation in the PB1-F2 of H5N1 (HK/97) and 1918 influenza A viruses contributes to increased virulence. *PLoS Pathog* **3**, 1414-1421 (2007).

12. Schmolke, M.*, et al.* Differential contribution of PB1-F2 to the virulence of highly pathogenic H5N1 influenza A virus in mammalian and avian species. *PLoS Pathog* **7**, e1002186 (2011).

13. Leung, B.W., Chen, H.L. & Brownlee, G.G. Correlation between polymerase activity and pathogenicity in two duck H5N1 influenza viruses suggests that the polymerase contributes to pathogenicity. *Virology* **401**, 96-106 (2010).

14. Song, M.S.*, et al.* The polymerase acidic protein gene of influenza a virus contributes to pathogenicity in a mouse model. *J Virol* **83**, 12325-12335 (2009).

15. Kim, J.H.*, et al.* Role of host-specific amino acids in the pathogenicity of avian H5N1 influenza viruses in mice. *J Gen Virol* **91**, 1284-1289 (2010).

16. Chen, G.W.*, et al.* Genomic signatures of human versus avian influenza A viruses. *Emerg Infect Dis* **12**, 1353-1360 (2006).

17. van Wielink, R.*, et al.* Mutations in the M-Gene Segment Can Substantially Increase Replication Efficiency of NS1 Deletion Influenza A Virus in MDCK Cells. *Journal of Virology* **86**, 12341-12350 (2012).

18. Murakami, S.*, et al.* Growth determinants for H5N1 influenza vaccine seed viruses in MDCK cells. *J Virol* **82**, 10502-10509 (2008).
